# The Baby Hearts Study – a case-control methodology with data linkage to evaluate risk and protective factors for congenital heart disease.

**DOI:** 10.23889/ijpds.v4i1.582

**Published:** 2019-04-08

**Authors:** N McCullough, H Dolk, M Loane, BM Lagan, F Casey, B Craig

**Affiliations:** 1 Institute of Nursing and Health Research, Ulster University, Newtownabbey, UK; 2 Paediatric Cardiology Department, Royal Belfast Hospital for Sick Children, Belfast, UK

## Abstract

**Introduction:**

The Baby Hearts study aimed to investigate risk and protective factors for congenital heart disease (CHD), and to investigate the health behaviours of a representative sample of pregnant women in Northern Ireland.

**Objectives:**

We describe and evaluate the population-based case-control design enhanced with data linkage to administrative health data.

**Methods:**

Cases (mothers of babies with CHD, n=286) were recruited following diagnosis prenatally or postnatally. Controls (mothers of babies without CHD, n=966) were recruited at 18-22 weeks gestation, from all women attending each maternity unit during a designated month. Hybrid data collection methods were used, including a self-administered iPad/postal questionnaire, and linkage to maternity and prescription records.

**Results:**

Refusal rates were low (8%). iPad questionnaire completion at clinic or home visit had high acceptability whereas postal questionnaires were poorly returned leading to a further 9-10% loss of eligible cases/controls. In total, 61% of eligible cases and 68% of eligible controls were recruited, closely representative of the Northern Ireland population, with no evidence of selection bias. Of those recruited, 97% gave consent for linkage to medical records. Thirty-three percent of women had an unplanned pregnancy and 76% suspected they were pregnant by 5 weeks gestation, with no significant differences between cases and controls. There was considerable discordance between self-report, maternity and prescription records regarding medications obtained/taken in the first trimester, but no evidence of differences between cases and controls that would indicate substantial recall bias. Although there was high concordance between self-report and maternity records regarding folic acid supplementation, cases had significantly lower concordance than controls.

**Conclusions:**

Our results suggest hybrid data collection approaches are a useful way forward for aetiological studies to reduce responder burden and address and estimate recall bias, and that the Baby Hearts study protocol is suitable for replication in other populations, modified to the local context.

## Introduction

Congenital heart disease (CHD) accounts for nearly one third of babies with major congenital anomalies diagnosed prenatally or in infancy in Europe, with an average prevalence of 8 per 1,000 births, varying between countries [1]. Whilst there have been significant advances in identifying the role of genetic factors, slow progress has been made recently in understanding environmental risk factors for CHD [2,3]. Previous large case-control studies have included the Baltimore-Washington Infants Study [4] and the United States National Birth Defects Prevention Study [5], but no large aetiological studies have been published from Europe. New methods of data collection have meanwhile become available for case-control studies, including linkage to health records, to increase their cost-effectiveness and potentially tackle sources of bias [6].

The aim of the Baby Hearts study was to investigate environmental risk and protective factors for CHD. The case-control study was conducted in Northern Ireland and focused particularly on common exposures where there is scientific uncertainty about their relationship to CHD risk, including periconceptional folic acid [7,8], maternal smoking [9,10], maternal obesity [11,12], and maternal depression and use of antidepressants [13,14]. Primary data collection from women, enabled by iPad questionnaires, is enhanced by linkage to electronic healthcare records (prescription data and maternity data) and area-based deprivation data. A secondary aim was to investigate the health behaviours of a representative sample of pregnant women (the control population) in the periconceptional period.

In this paper, we describe and evaluate the design and methodology of the Baby Hearts Study using hybrid data collection methods. We assess the feasibility of recruiting cases and an unbiased population sample of cases and controls; the acceptability, advantages and limitations of data collection from mothers via iPad questionnaire and potential for recall bias; and the feasibility and added value of data linkage with health records to reduce responder burden and reduce recall bias. We thus propose a protocol which can be reused in future (modified to local contexts). We also establish baseline information on population characteristics and data quality for the interpretation of the results.

## Methods

### Design and Setting

A population-based case-control study was conducted. Northern Ireland has approximately 24,000 annual births, with maternity care free to all resident women and paediatric cardiology care delivered by a single specialist centre within the geographic region. All babies with CHD suspected prenatally or postnatally are referred to this centre. Termination of pregnancy is not legal in Northern Ireland.

### Definition of cases and controls and eligibility criteria.

Cases were babies diagnosed with CHD (prenatally or up to 6 months of age) born to mothers resident in Northern Ireland, diagnosed by the regional specialist centre and recorded in the Heartsuite database (a clinical information management system used by paediatric cardiology services). A diagnosis of CHD is made after babies have undergone a range of tests, including an echocardiogram which is considered as the gold standard. Stillborn babies with CHD were eligible for inclusion if diagnosis had been made prenatally. All cases of CHD were further classified by the paediatric cardiologists (FC and BC), based on diagnostic information extracted from the clinical database, to categories proposed by Houyel and colleagues [15]. This system groups cases into one of 10 main categories based on anatomical and clinical criteria.

CHD recruitment excluded patent ductus arteriosus linked to prematurity, patent foramen ovale, and CHD associated with Down Syndrome. CHD associated with other genetic syndromes are to be excluded at data analysis stage. Controls were babies without CHD with mothers resident in Northern Ireland at time of recruitment. No other health restrictions were placed on controls. Cases and controls were included if their mothers could read and understand English or Polish (the most frequent language among non-English speakers in Northern Ireland) and were aged at least 17 years at time of recruitment.

### Identification, Consent and Recruitment of cases

Cases were identified, consented and recruited from September 2014 to February 2017. Eligible babies with CHD were identified by clinical staff screening in/outpatient lists and the clinical database. Mothers of babies with CHD identified prenatally were given information by the Fetal Cardiology Nurse, and if interested were contacted by a researcher, who arranged to meet at a future hospital appointment. For babies identified postnatally, information was sent by post informing case mothers that a researcher would invite them to participate at their next hospital appointment. For those women whose babies had less frequent appointments or attended outreach clinics, the study questionnaire was sent by post, followed by a phone call. Due to poor response for postal questionnaires, women were later offered a home visit. Thirteen cases who were diagnosed after 6 months of age were recruited, where the defect was severe enough that an earlier diagnosis would have been expected.

### Identification, Consent and Recruitment of Controls

Controls were identified, consented and recruited during the period June 2014 to February 2016. Recruitment of control mothers used a “one month per health unit” intensive approach, with every eligible woman attending for her first anomaly scan (at 18-22 weeks gestation) during that month eligible for recruitment by the researcher. Seventeen health units performed fetal anomaly scans throughout Northern Ireland. This strategy was designed to result in a representative population sample, and to minimise disruption and simplify access in maternity units. Posters were displayed within each unit, stating the designated month for recruitment in that particular unit. Midwives were asked to hand patient information leaflets to all women attending for their antenatal booking appointment between 8-14 weeks gestation so that they would expect the researcher in the designated month, but during recruitment, women were also given time to read the information leaflets as some women did not remember receiving it or had moved between units. The patient information leaflet was developed with the input of clinical staff and patient representatives, and included positive pictures of children of various ages with CHD whose mothers had given consent for their images to be used.

Women completed the questionnaire by iPad before or after their scan or they could choose to take a paper questionnaire home instead (questionnaires in Polish were only available on paper) and for two weeks, follow-up reminder calls were made. Where participants initially took part as a control mother, but their baby was subsequently diagnosed with CHD, mothers were asked for new consent for inclusion of their baby as a case. As an incentive to participate and token of appreciation, all participants (cases and controls) were offered a gift token for £10 to be redeemed in a number of online and high street shops.

### Exposure Information

Women were asked to complete a self-report questionnaire, and to give permission for linkage of these questionnaires to medical records.

#### Self-report questionnaire

Topics included sociodemographic information, questions about when the woman suspected and confirmed she was pregnant, folic acid supplementation, diet (focusing on folate rich foods), maternal smoking and alcohol use, exercise, exposure to stressful life events, maternal and paternal health conditions, medication use and exposure to a variety of substances at home and work [Appendix 1]. Sources for questions included the Baltimore Washington Infant Study [4], the National Birth Defects Prevention Study (NBDPS) [5], the Avon Longitudinal Study of Parents and Children (ALSPAC) [16], the Pregnancy Risk Assessment Monitoring System (PRAMS) studies [17], and a case-control study of gastroschisis [18], with additional questions formulated by the study team.

Although paternal factors were not the subject of study, a few questions were included about the father/partner’s health, to take the focus off the woman in terms of potential guilt.

The periconceptional period of interest was defined as three months before conception and the first three months (12 weeks) of pregnancy. The preconceptional period is important to ascertain “normal” behaviours until women find out they are pregnant, and to allow for the longer acting exposures. “Postconceptional” was defined as the first three months of pregnancy.

An iPad application was programmed with touch screen functionality for data entry and a skip logic function that automatically hid questions not relevant to the mother (based on her previous responses). Prompts informing participants of the number of skipped questions and taking them back to them were used to reduce the proportion of missing responses. To facilitate recall, a timeline was created, visible at the bottom of each page, to show women the calendar dates corresponding to the three months before and after conception. This was estimated based on their answers to a question regarding their current gestational age (or for postnatal cases, estimated due date), combined with the current date. The iPad/tablet had the additional advantages of confidentiality allowing honesty, and direct coding of answers.

The questionnaire was designed to be as short as possible in order to ensure a high response and completion rate. Pilot studies indicated that women preferred completion to take no longer than 15-20 minutes. Depending on the complexity of exposures reported by the woman, time taken to complete ranged from 12 to 25 minutes. The questionnaire and information leaflets were reviewed by the study’s external advisory committee which included a mother of a child with CHD. The App will be made freely available at completion of the study.

#### Maternity records

The Northern Ireland Maternity System (NIMATS) is a web based information system that collects antenatal, intranatal and postnatal data on all women in receipt of maternity care in NI. Where women gave their consent, information recorded at the ‘booking appointment’ at 10-12 weeks was taken from NIMATS. Information collated included parity, Body Mass Index, date of Last Menstrual Period (LMP), nausea experience, medication use during pregnancy, pre and post-conceptional folic acid use, smoking habits and alcohol consumption, diabetes and other maternal chronic diseases.

#### Pharmacy records

Information on prescriptions issued by General Practitioners and dispensed by pharmacists in Northern Ireland are stored in the Enhanced Prescribing Database. Information includes the date the prescription was redeemed, non-proprietary and proprietary name of the medication, and dosage and quantity dispensed. We requested an extract of information on all medications from British National Formulary chapters 2 (Cardiovascular system); 3 (Respiratory system including antiasthmatics chapters 3:1:1 and 3:2:2), 4 (Central nervous system including antidepressants chapters 4:3:3 and 4:3:4), 5 (Infections including antibiotics chapters 5:1:1 to 5:1:13), 6 (Endocrine system) and 9 (Nutrition and blood) that were prescribed during the periconceptional period, where maternal consent had been given, using the participant’s unique Health and Social Care number. The timing of prescription during (or before) pregnancy was estimated using the LMP recorded in the maternity records for cases and controls.

#### Paediatric cardiology records

Paediatric cardiology records were accessed for type of CHD and age at diagnosis.

#### Area-based data

An area based deprivation index was linked according to postcode of residence of the mother in the first three months of pregnancy [19]. In future, other area based data can be linked, including air pollution data.

### Sample size

The sample size target (80% power, significance level 5%) was 400 CHD cases, with ratio of 2 controls to 1 case, to detect Odds Ratios between 1.5 and 2 for common exposures of 10% to 25% prevalence in the pregnant population as predicted for the main exposures of interest. We recognised that sample power for specific CHD subgroups would be limited. Our achieved sample size was over the target for controls, and under the target for cases (Figure 1a). For controls, extra recruitment was due to dropping of the requirement for all women to have received the information leaflets at booking, while preserving the monthly recruitment plan (Figure 1b). For cases, we overestimated the participation rate and underestimated the proportion who would be diagnosed at over 1 month of age, curtailing recruitment of later births.

**Figure 1a: Flow diagram of recruitment for cases:**
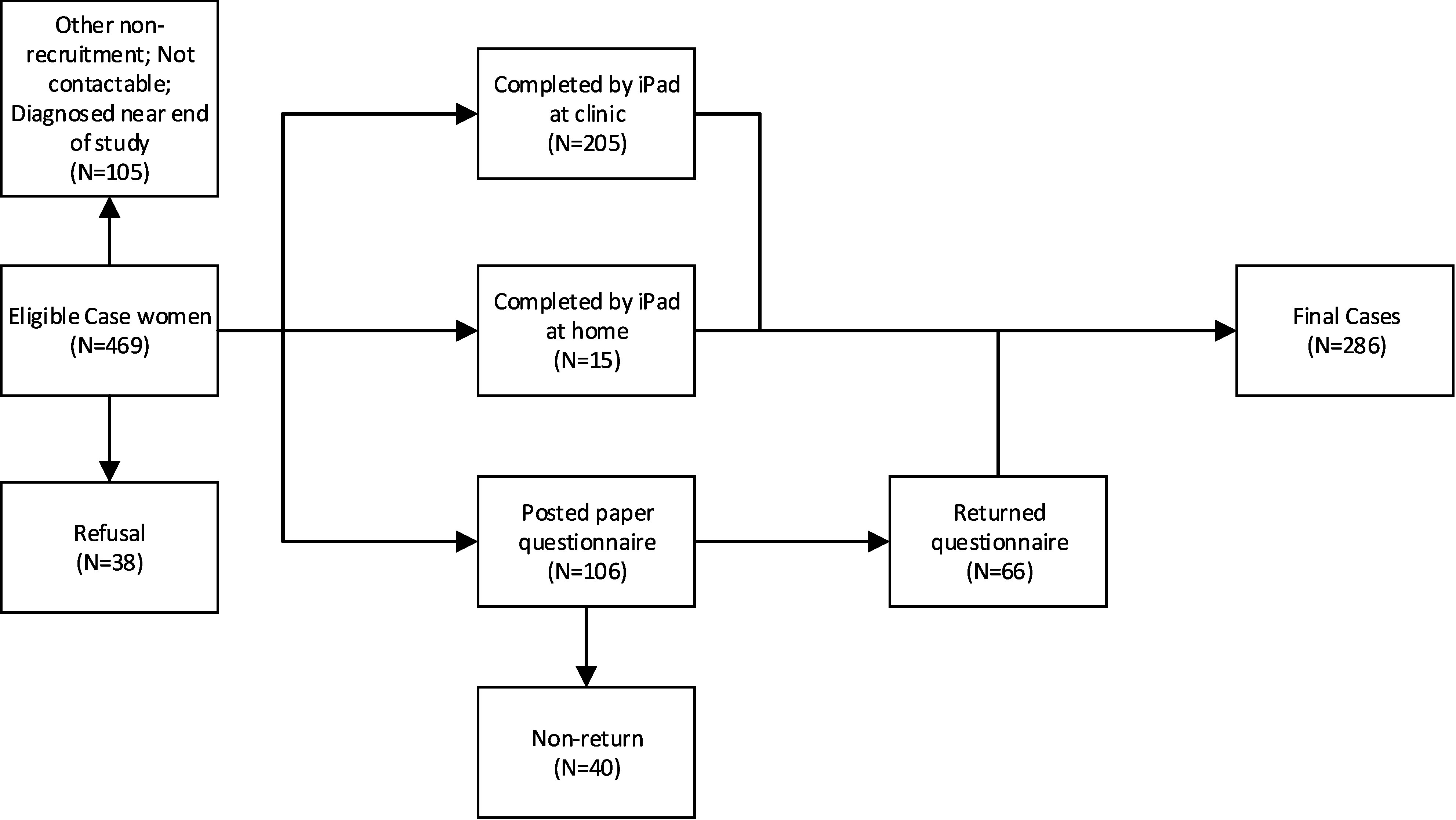
*N= 469 includes N=12 originally identified as controls

**Figure 1b: Flow diagram of recruitment for controls:**
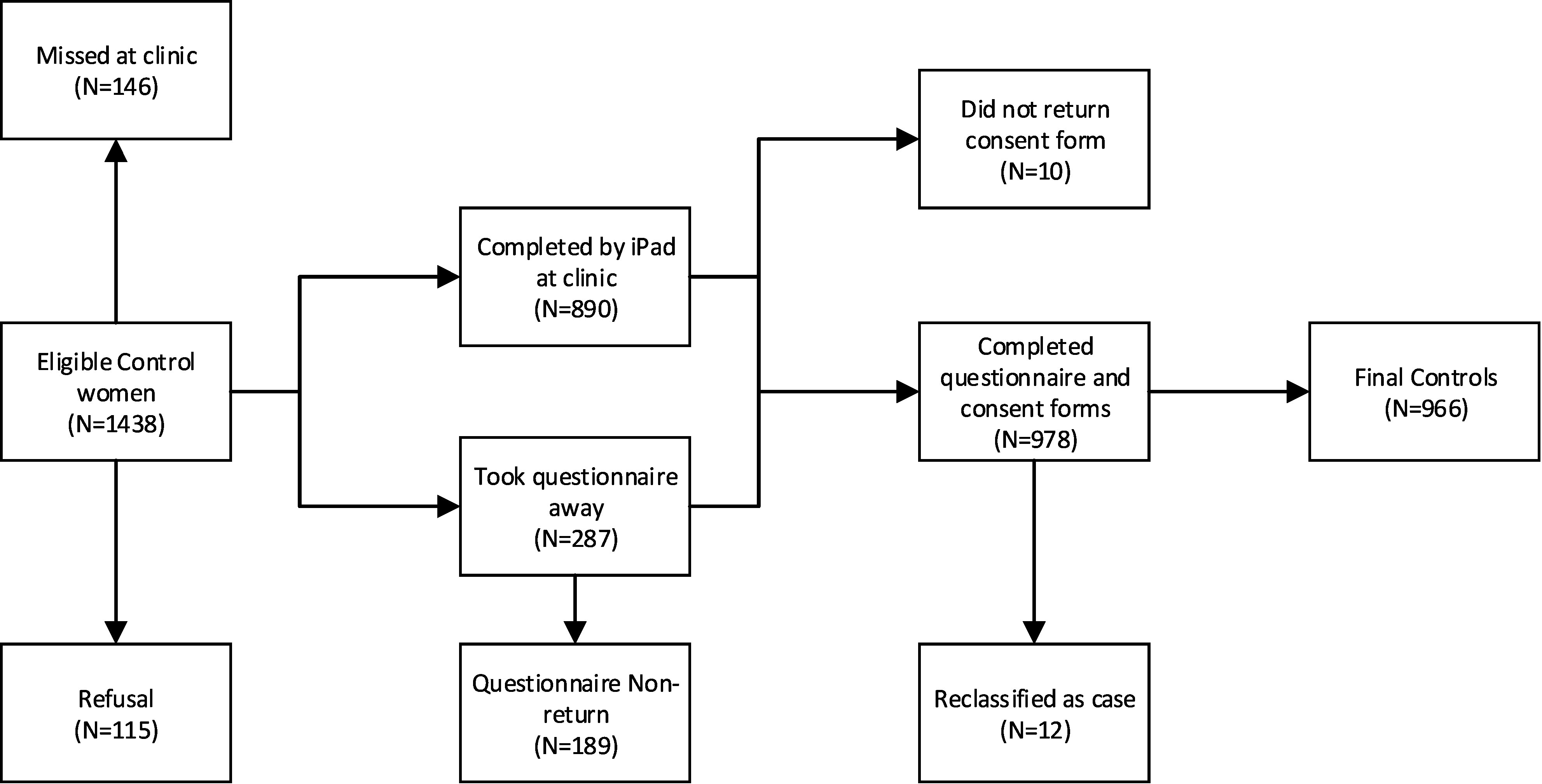


### Statistical Analysis

Potential recruitment and recall bias was assessed with the chi square test comparing controls and the general population, and comparing controls and cases. Fisher’s exact test was used where one or more cell sizes were below 5. Differences in the number of missing question responses per woman were examined using the Wilcoxon rank-sum (Mann-Whitney) test. Analysis was carried out using STATA version 12.

### Ethics and governance

Ethics approval was obtained from the Office for Research Ethics Committees Northern Ireland. Research and Governance approval was obtained from each Trust. Signed informed consent was obtained from all women.

## Results

### Recruitment rates

The final sample size was 286 cases and 966 controls. Sixty-one percent of eligible cases and 68% of eligible controls were recruited (Figures 1a & 1b). The refusal rate was low for both groups (8% cases, 8% controls). Non-return of postal questionnaires accounted for 9% of eligible cases, and 13% of eligible controls. Other reasons for non-participation were mostly logistical (Figures 1a & 1b). Twelve controls transferred to case status, slightly more than the number expected to do so (expectation was 8 per 1,000 * 978 = 8). Most completed the questionnaire using the iPad (cases: 77%; controls: 90 %).

The most common categories of CHD were Ventricular Septal Defects (28%);anomalies of the extrapericardial arterial trunks (including coarctation of the aorta) (26%); and anomalies of the ventricular outflow tracts which include Tetralogy of Fallot and Transposition of the Great Arteries (15%).

### Demographic and periconceptional characteristics and potential for recruitment bias

Of the key sociodemographic and exposure characteristics of controls, the only significant difference compared with the general population was country of birth of mother, with less study women born outside the United Kingdom (UK) UK/Ireland (7% vs 10%, Table 1). Cases and controls were comparable except for a tendency for cases to be less educated (P=0.009, Table 1). Comparing controls who completed questionnaires by iPad or on paper (data not shown), sociodemographic characteristics were comparable, except that those from rural areas were more represented among iPad completers (37.9% vs 22.6%, P<0.001) and those born in countries outside the UK/Ireland were more represented among paper questionnaire completers (21.7%) than iPad completers (5.4% P<0.001).

**Table 1: Distribution of sample by key demographic variables, and comparison with population information where available.** ^(a)^Public Health Agency. Children’s Health in Northern Ireland. [Internet]. 2016. Available from: http://www.publichealth.hscni.net/sites/default/files/RUAG report 2015-16 - Children’s Health in NI - FINAL REPORT - May 2016.pdf (Notes:1.This report uses birth from a range of sources resulting in differences in totals; 2.Births reported are for one year in total distributed over 2 calendar years. See report for full details)

	Cases N=286		Controls N=966		P value for cases vs controls	Population(^a^) 2014-2015		P values for controls vs Population

	N	%	N	%	P	N	%	P

**Age Mother**								
≤24	45	15.7	158	16.4	P=0.43	4153	17.0	P=0.42
25-29	76	26.6	256	26.5		6619	27.1	
30-34	93	32.5	350	32.2		8220	33.7	
35+	72	25.2	202	20.9		5405	22.1	
**Level of Education**								
Left after compulsory education completed	78	27.3	193	20.0	P=0.009	-	-	
Higher secondary school/Technical college	107	37.4	351	36.3		-	-	
University degree	100	35.0	421	43.6		-	-	
Missing	1	0.4	1	0.01		-	-	
**Deprivation[^19^]**								
Quintile 1 Most deprived	72	25.8	196	20.3	P=0.39	5612	23.4	P=0.45
Quintile 2	55	19.2	200	20.7		5313	22.1	
Quintile 3	54	18.9	212	22.0		5155	21.5	
Quintile 4	51	17.8	191	19.8		4749	19.8	
Quintile 5 Least deprived	41	14.3	147	15.2		3571	14.9	
Missing	13	4.6	20	2.1				
**Area of Residence**								
Urban	175	61.2	576	59.6	P=0.75	-	-	
Rural	98	34.3	352	36.4		-	-	
Missing	13	4.6	38	3.9		-	-	
**Country of birth of mother**								
Northern Ireland	233	81.5	791	81.9	P=0.64	20129	82.5	P=0.02
Other UK/Ireland	17	6.0	74	7.7		1799	7.4	
All other countries	20	7.1	64	6.6		2469	10.2	
Missing	16	5.6	37	3.8		0	0.0	
**Smoking at Booking**								
Yes (1+)	40	14.0	109	11.3	P=0.12	3500	14.5	P=0.06
Missing	37	12.9	78	8.1		-	-	
**Planned pregnancy (self-report)**								
Yes	176	61.5	642	66.5	P=0.12	-	-	
No	109	38.1	320	33.1		-	-	
Missing	1	0.4	4	0.4		-	-	
**Maternal BMI at booking**								
Underweight (<18.50)	5	1.75	11	1.2	P=0.93	479	2.0	P=0.17
Normal (18.50-24.99)	130	45.5	458	47.3		11613	48.6	
Pre-obese (25.00-29.99)	81	28.3	274	28.4		7026	29.6	
Obese I (30.00-34.99)	38	13.3	116	12.0		2954	12.4	
Obese II (35.00-39.99)	13	4.6	51	5.3		1221	5.1	
Obese III (>=40.00)	9	3.2	33	3.4		579	2.4	
Missing	10	3.5	23	2.4		313	1.3	

Thirty-three percent of mothers responded “I was not planning to become pregnant”, with no significant difference between cases and controls (P=0.12, Table 1). Figure 2 shows the gestational age at which women suspected they were pregnant, first saw a health professional, and had a “booking appointment” with a midwife. By 5 weeks’ gestation, 76% (n=951) of both cases and controls suspected they were pregnant. The curves for both cases and controls are very similar.

**Figure 2: Cumulative frequency of gestational age when pregnancy first suspected, at first contact with a Health Care Professional (HCP) about the pregnancy, and at booking appointment with midwife, for cases and controls.**
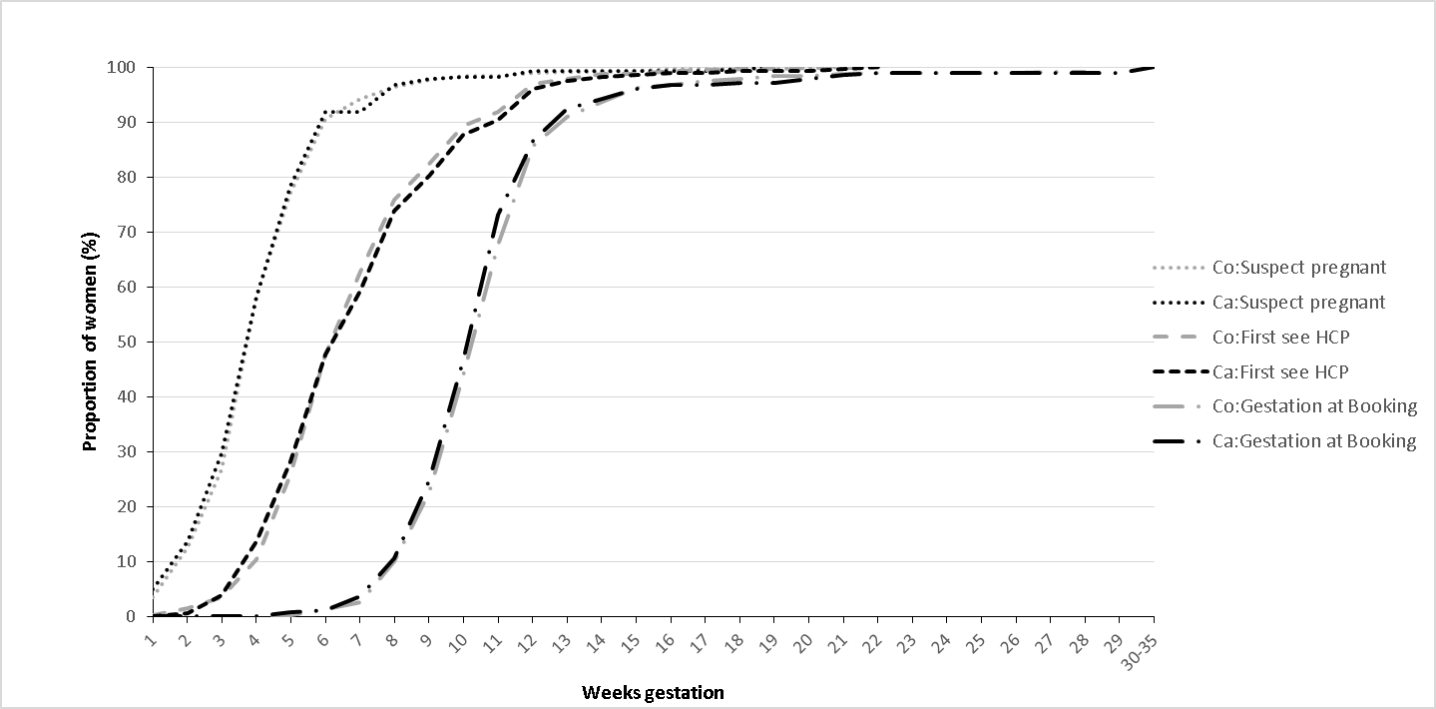
Note: Co = Controls; Ca = Cases

### Time of diagnosis of CHD and questionnaire completion.

Twenty-eight percent of cases were diagnosed prenatally, 30% before they were one month old, and 42% thereafter. Cumulatively, 16% of the case sample had completed the questionnaire during the prenatal stage, 20% by 1 month of age, 58% by 6 months, and 88% by 1 year. The median time between pregnancy onset (LMP) and survey completion for cases was 56 weeks. Controls completed the questionnaire at a median of 20 weeks’ gestation (range 18-34 weeks’ gestation).

### Permission for data linkage and follow up

Overall a high proportion of the sample gave permission to access additional medical information (97-98%) and to be followed up for future studies (95%). For cases, the mode of questionnaire completion did not influence the decision to allow access to medical records (97-98%). Controls who completed the paper version of the questionnaire were less likely than those who completed iPad questionnaire to give permission for all types of data access (91-92%, P<0.001 for each type) and for follow up for future studies (73%, P<0.001).

### Missing data

The proportion of missing data for the questionnaire for most items ranged from 0% to 3%. The only exceptions were that while women reported overall levels of alcohol, responses to questions about specific drinks and drink sizes were missed for 5-9%.

The total number of missing items per person ranged from zero (63%) to 56 (0.2%). This did not significantly vary between cases and controls (z= -1.952, P=0.051) but did vary by mode of completion with paper completers having a significantly higher rate of missing answers (z = -16.327, P<0.001). Of women who had given consent, we were unable to access the full maternity data for 0.6% (n=8) of women from maternity records, and 1.6% (n=15) from prescription data.

### Data linkage and concordance between sources of data for medication use and pregnancy planning

Concordance between data sources was examined for antiasthmatics, antidepressants and antibiotics. Maternity data reported considerably lower frequencies of exposure for all three medication types, but a high proportion (76-86%) of maternity reports were confirmed by the self–reported questionnaire (Table 2). The pattern of concordance between self-report and prescription data differed according to type of medication. For antidepressants, 84% of self-reported exposures were confirmed by postconceptional prescription among controls, rising to 91% when considering prescriptions in the periconceptional period (Table 2). The remaining discordance is explained by women self-reporting diazepam as an antidepressant which was not included in the SSRI/SNRI-based prescription measure. For antibiotics and antiasthmatics, the confirmation rates were lower (Table 2), but increased somewhat if periconceptional prescriptions were considered. There were no statistically significant differences between cases and controls (p>0.05 for all comparisons).

**Table 2: Agreement between self-report data (questionnaire) and administrative data (maternity data and prescription records) for medication use, preconception folic acid supplements, and unplanned pregnancy.** Note: Pre-conception: three months before pregnancy; Postconception: first three months of pregnancy; Periconception: three months before and first three months of pregnancy

Type of Exposure	Control/Case	Number exposed to medication as reported by three data sources			Proportion of exposure reports of one data source confirmed by another source.			

		Self- report - taken in first trimester	Maternity data -reported at 10-12 weeks gestation	Prescription data - post conception (periconception)	Self- report confirmed by maternity data	maternity data confirmed by self- report	Self-report confirmed by prescription post conception (peri-conception)	Post conception (periconception) prescription confirmed by self-report

		N	N	N	%	%	%	%

Antidepressants	Controls	32	21	50 (68)	50.0	76.2	84.4 (90.6)	54.0 (42.6)
	Cases	7	4	16 (25)	42.9	75.0	71.4 (100.0)	31.3 (28.0)
Antibiotics	Controls	105	13	140 (222)	10.5	84.6	59.0 (67.6)	44.3 (32.0)
	Cases	27	4	36 (55)	3.7	25.0	48.1 (55.6)	36.1 (27.3)
Antiasthmatics	Controls	58	28	43 (64)	41.4	85.7	53.4 (75.9)	72.1 (68.8)
	Cases	13	8	13 (19)	46.2	75.0	61.5 (84.6)	61.5 (57.9)
Preconception Folic acid	Controls	347	337	-	81.3	83.7	-	-
	Cases	86	92	-	79.1	73.9	-	-
Unplanned pregnancy	Controls	301	227	-	70.4	93.4	-	-
	Cases	94	66	-	63.8	90.9	-	-

Postconception (first three months) prescription data reported higher numbers of women receiving prescriptions than self-reported exposure for antidepressants and antibiotics, but lower for antiasthmatics. Proportions of postconceptional prescriptions which were confirmed by self-reported exposure among controls varied between 44% for antibiotics and 72% for antiasthmatics, with no significant differences between cases and controls. Agreement was lower if periconceptional prescriptions were considered. Agreement was high between maternity data and self-report as to whether folic acid had been started preconceptionally (Table 2). However, the proportion of women whose maternity report of folic acid was confirmed by self-report was significantly higher for controls (84%) than cases (73%, p=0.03). Women were more likely to have self-reported that they had not planned to become pregnant than was recorded in the maternity data (Table 2), but maternity data was generally confirmed by self-report, and there were no significant differences in proportion between cases and controls.

## Discussion

### General considerations

We have developed a methodology and tools which could be a useful basis for other studies and eventual meta-analysis, as there is a huge need for more epidemiological evidence about the environmental causes of congenital anomalies, and particularly congenital heart disease, as a basis for primary prevention [20]. Our experience suggests that a hybrid means of data collection, employing technologies such as iPad for self-reported data, with linkage to electronic healthcare databases for information collected (prospectively) by health professionals is a promising way forward. Linkage to health records means that information to be gained by self-report can be kept to a minimum of information not recorded in health records (such as education, over the counter medication and prepregnancy lifestyle factors), thus reducing responder burden and improving participation. Linking prospectively collected information also means that the potential for maternal recall bias in case-control studies can be diminished, or its extent estimated. The final hybrid dataset becomes a very rich dataset.

We found high levels of permission for record linkage. However, controls who had chosen to take the paper questionnaire for postal return were a little less likely to give permission for record linkage, perhaps because they were in general less keen on participation, or less contact with the researcher generated less trust, or consultation with family members who had not met with the researcher led to lower levels of trust. This suggests that permission is context dependent, which is important to take into account in planning hybrid data linkage research. Our control population was a representative sample of the Northern Ireland population as judged by key sociodemographic characteristics, except for the slightly lower participation of those born outside UK/Ireland (partly by design due to budget limitations for language translation). We are therefore able to use the information gathered to analyse the health conditions of the pregnant population in Northern Ireland, adding to the cost-effectiveness of the study.

One third of mothers reported that they had not planned to become pregnant (a slightly higher proportion than estimated from maternity records), and one quarter had not suspected they were pregnant before 6 weeks gestation. To our knowledge, these are the first population-based reports of the timing of pregnancy recognition, and these data enable us to investigate in the control population timing of health behaviour change and change after contact with a health professional. Since the major development of the fetal heart occurs when women may not know they are pregnant, these results also confirm the importance of asking about women’s usual prepregnancy exposures as well as change during the first trimester when studying the aetiology of congenital heart disease, and planning public health interventions to prevent birth defects.

### Recruitment and recruitment bias

Although there have been concerns about the low levels of recruitment currently achieved in perinatal studies, we found our recruitment strategy for control women to be successful: recruitment by a researcher within the maternity environment, a designated month for recruitment per unit (rather than continuous sampling across the entire population), use of an iPad to increase ease and enjoyment of participation, having a short questionnaire and an attractive information leaflet, and offer of a small incentive/token of gratitude. Postal questionnaires were poorly returned, and phone contact with women tended to be difficult to establish. We did not test the option of web-based [21,22] or App questionnaires to complete at home, however our experience suggests completion at time of recruitment leads to higher response. The rate of refusal was low. Most non-recruitment was for logistical reasons and the final sample remained representative of the Northern Ireland pregnant population.

Comparison of sociodemographic characteristics between cases and controls revealed little indication of potential selection bias, except in relation to maternal education, which may be a risk factor for congenital heart disease. Anecdotally, we were aware of a few mothers with eligible cases who did not want to participate due to feelings of potential guilt an issue that has not been addressed in the methodological literature which tends to focus on maternal recall bias.

Contrary to our expectations, the recruitment of cases proved more difficult than recruitment of controls, due to the logistical difficulties of meeting women at their hospital appointments, and poor return of postal questionnaires. We found the investment of the researchers time in home visits in the later part of the study was not disproportionate compared to trying to meet mothers at clinics, and in future, we would recommend this method of recruitment. We recruited less cases prenatally, and less in the early neonatal period, than we had expected. The US National Birth Defect Prevention Study has also noted late recruitment of cases [23] and this is a key problem in case-control studies of congenital anomalies [24] especially of congenital heart disease where many diagnoses are made after the neonatal period. Although matching controls to cases by age at recruitment would be theoretically less prone to information bias, we had no access as researchers to a suitable population-based sampling frame to achieve this, nor a recruitment context for controls similar to the maternity unit opportunity. The key concern then is whether there is evidence of information bias relating to maternal recall to outweigh the advantages of our recruitment method.

### Assessment of recall bias

Recall bias, where mothers of cases with congenital anomalies are more motivated to recall exposures than mothers of controls, has been much discussed in the literature, with assessments varying as to its possible strength, and possible ways to mitigate it [25,26]. In our study, there is an additional recall bias issue - the longer period of recall for cases than controls leading to potential poorer recall by case mothers. Case mothers were much further beyond their early pregnancy exposures than controls, and had experienced much in between with the birth of a baby with CHD. The iPad was a useful tool on which to establish, display and reinforce the early pregnancy time window to aid recall. We found remarkable similarity between cases and controls in the curves of when women recalled that they had become aware of their pregnancy and sought health professional advice, reinforcing confidence in the recall of key events. In general, levels of concordance between recalled medication exposures and medical records were slightly lower for cases, which might suggest slightly poorer recall of exposure or exposure timing over the longer recall period, or a tendency for cases to over-report, but this was not statistically significant. The only significant difference was that maternity reports of folic acid intake were less likely to be confirmed by the self-report of cases than controls, consistent with the effect of a longer recall period or over-reporting by cases. Lack of evidence of systematic recall bias leads us to conclude that comparability of timing of interview of cases and controls was worth sacrificing to gain high recruitment and representative population samples. Nevertheless, the potential for some recall bias needs to be taken into account in the interpretation of results.

### Concordance of self-report, maternity data and prescription data

There is no gold standard for measuring medication exposure in pregnancy, yet the issue of medication safety in pregnancy is extremely important and under-researched [27]. Our data shed light on the advantages and disadvantages of different data sources, showing this to be dependent on medication type. The maternity database (NIMATS) was a poor source of data, with low sensitivity for exposures compared to self-report, for all medication types. This is probably because the maternity database system only asks at the time of the booking appointment about current medication intake. The particularly low sensitivity for antibiotics as opposed to chronic disease medications reflects this. We concluded that maternity data, for our study, had little extra value, and that if maternity databases are to become an important source of data for pregnancy-related medication safety studies, more emphasis is needed on recording early pregnancy exposures.

For antidepressants, retrospective self-report was very likely to be confirmed by prescription data. The proportion of postconceptional prescriptions confirmed by self-report was however not high. The potential reasons for a woman not self- reporting antidepressant exposure where the postconceptional prescription data suggests it has been prescribed include the woman not taking the medication because it is her first prescription and she decides not to, the woman not taking the medication because she is concerned about its safety in pregnancy, the woman obtaining the medication for others (known to occur with antidepressants in our population), poor recall about exposures prior to pregnancy recognition, or reluctance to declare exposure. We concluded that self-report was the best primary measure of exposure, with secondary sensitivity analysis using postconceptional prescription data to explore potential for recall bias.

The situation regarding antiasthmatics (which are all prescription-only in Northern Ireland) is different – women are advised not to discontinue antiasthmatics in pregnancy [28], and many antiasthmatics are taken on an intermittent rather than chronic basis. This means that prescription dates are a poorer guide to timing at use [29], but also that recall of exact timing of use in relation to recognition of pregnancy may be more difficult. The proportion of self-reported antiasthmatics confirmed by periconceptional prescriptions was lower for antiasthmatics than for antidepressants, probably indicating that some prescriptions had been obtained before the periconceptional period. On the other hand, compared to antidepressants, prescriptions were more likely to be confirmed by self-report, probably reflecting the fact that women did not stop using antiasthmatics that they had been prescribed [28]. We concluded again that self-report is the most appropriate primary measure, with prescription data a secondary measure less accurately describing first trimester exposure, but guarding against recall bias.

Acute use of medications such as antibiotics present a different problem. Here we saw the greatest discordance between self-report and prescription data, probably reflecting both difficulties recalling short exposures and exact timing, as well as population behaviour where antibiotics are picked up from the pharmacy (prescriptions in Northern Ireland are free) but not necessarily used, or antibiotics are not used at the time of prescription, or there is reluctance to use in pregnancy despite medical advice. In a study in the Netherlands [30], 82% of interviewed women who were prescribed antibiotics in the first trimester took their medicine, but it is not clear if the same applies to the Northern Ireland population. We concluded that in the absence of a reliable measure, both self-reported exposure confirmed by prescription, and postconceptional prescriptions could be analysed as alternative measures. Our data show that for medication safety studies, research is needed to understand the medication-taking behaviour of the pregnant population to inform exposure measurement.

## Conclusion

The strengths of the Baby Hearts Case Control study are its population-based representative sample via a novel sampling method, diagnostic accuracy regarding CHD through direct access to medical notes, and hybrid data collection of retrospective maternal interview and linkage with (prospective) maternity, prescription and other records to improve the richness and validity of data. A further strength is that it can address questions about the aetiology of CHD, as well as about the health-related behaviours of a representative sample of pregnant women. The main limitation of our study in Northern Ireland is the smaller than intended achieved sample size for cases, but we believe there is potential for better recruitment in future. Our iPad App will be available for use by others, and we hope that in future similar studies can be conducted and co-ordinated for meta-analysis.

## Appendices

Appendix 1 Extract of Northern Ireland Baby Hearts Study Questionnaire (iPad version)
