# Supplementary material for: The Baby Hearts Study – a case-control methodology with data linkage to evaluate risk and protective factors for congenital heart disease
Source: Int J Popul Data Sci. 2019 Apr 8;4(1):582. doi: 10.23889/ijpds.v4i1.582 (PMC7479919; doi:10.23889/ijpds.v4i1.582)
Supplement: Appendix 1 Extract of Northern Ireland Baby Hearts Study Questionnaire (iPad version) [file ijpds-04-582-s001.pdf]

Online Appendix 1:

Extract of Northern Ireland Baby Hearts Study Questionnaire (iPad version)

Notes:

This is an extract of the Northern Ireland Baby Hearts Study Questionnaire, shown here in order to visually demonstrate the iPad interface. There are 144 questions in total, not all of which are shown here.

Screen one (page 4) demonstrates the creation of the periconceptional time line.

Part one was designed to collect a range of demographic and pregnancy history information about the women. The iPad function also incorporated prompts to help women answer the questions (pages 4-5).

Part two asked women to report on a range of diet and exercise factors relevant to either the three months before pregnancy or during the first three months. This includes taking folic acid supplementation (page 6); consumption of a range of fortified foods including breads (page 7); cereals, cereal bars and spreads (not shown) and folate rich foods (page 8). This section also asked about the frequency of consumption of non-alcoholic beverages and the frequency of exercising before and after pregnancy (both not shown).

Part Three asked women to report on both their own (page 9) and the baby's fathers (page 10) history of chronic health conditions. Women were also asked to report if they had had any infections or fever or high temperature during the first three months of pregnancy (pages 10-11). This section also asked women to report on the use of a range of selected medications during the first three months of pregnancy, including information on timing of starting and stopping the medication (pages 11-12).

Part Four asked women to report on smoking and drinking behaviour in the three months before and the first three months of pregnancy. Only an extract is shown of the questions on smoking (page 12). Other questions asked more details on timing of change in smoking behaviour, if women had been in a smoking cessation programme, if they had been exposed to others smoking

behaviours and if they had used electronic cigarettes. Similarly, only an extract is shown of questions about alcohol use (page 13), with additional questions asked about frequency of intake of specific types of alcohol according to size of glass and alcohol units.

Part Five asked women to report on experience of a range of stressful life events (N=10) during the periconceptional period or more recently. Only an extract of the questions are shown here (pages 13-14). Women were also asked to report if they had felt stressed about any aspect of their pregnancy and if they felt they had received good social support from families and friends (page 15).

Part Six, not shown here, asked a range of questions about home, work and hobbies during the first three months of pregnancy. This included questions on accommodation, type of job (if employed), hours worked and any changes that occurred after finding out about the pregnancy. Additional questions also asked about frequency of contact with a range of personal care and cleaning products, and direct or indirect engagement in a range of activities in the house or garden.

Part Seven, not shown here, asks all women to report on family history of congenital heart disease. This section also provided the women with an opportunity to tell us about any worries or concerns that they may have had about their pregnancy. This was a free-text function.

The last section of the questionnaire (page 15) thanked women for taking part in the study and also identified the number of missed questions. Women were given the option to view and complete these items or finish the questionnaire.

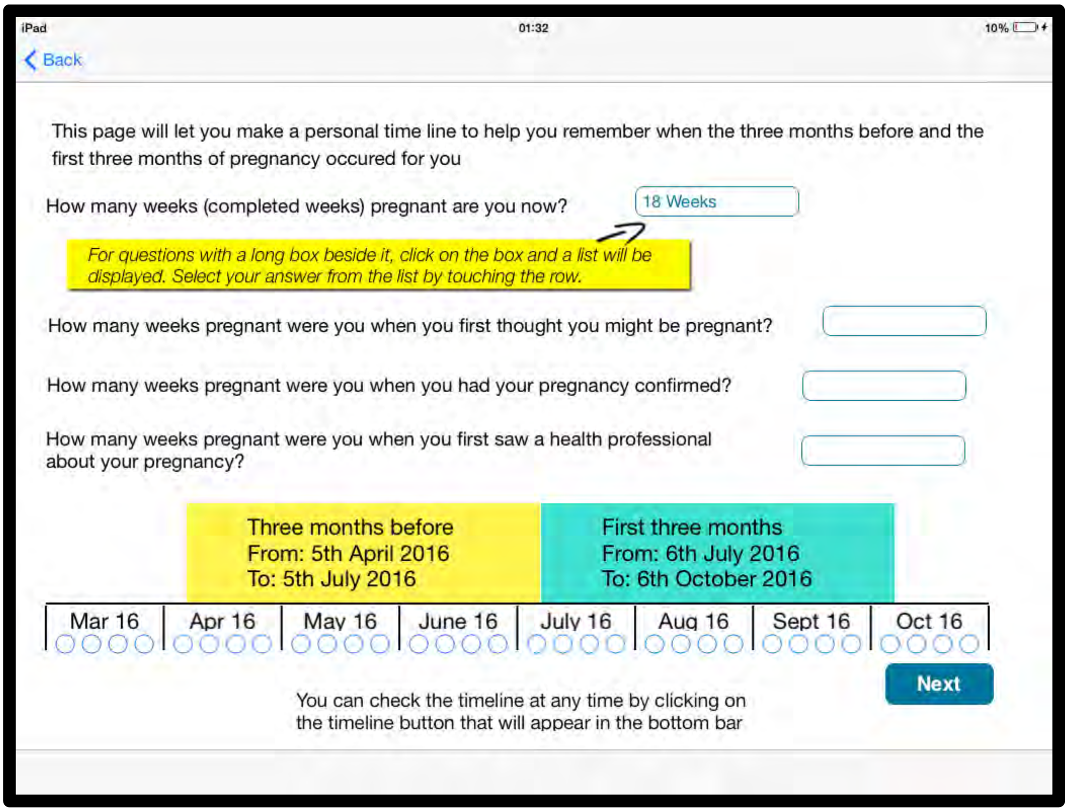
iPad
01:32
10%
Back
This page will let you make a personal time line to help you remember when the three months before and the first three months of pregnancy occured for you
How many weeks (completed weeks) pregnant are you now?
18 Weeks
For questions with a long box beside it, click on the box and a list will be displayed. Select your answer from the list by touching the row.
How many weeks pregnant were you when you first thought you might be pregnant?
How many weeks pregnant were you when you had your pregnancy confirmed?
How many weeks pregnant were you when you first saw a health professional about your pregnancy?
Three months before
From: 5th April 2016
To: 5th July 2016
First three months
From: 6th July 2016
To: 6th October 2016
Mar 16
Apr 16
May 16
June 16
July 16
Aug 16
Sept 16
Oct 16
Next
You can check the timeline at any time by clicking on the timeline button that will appear in the bottom bar

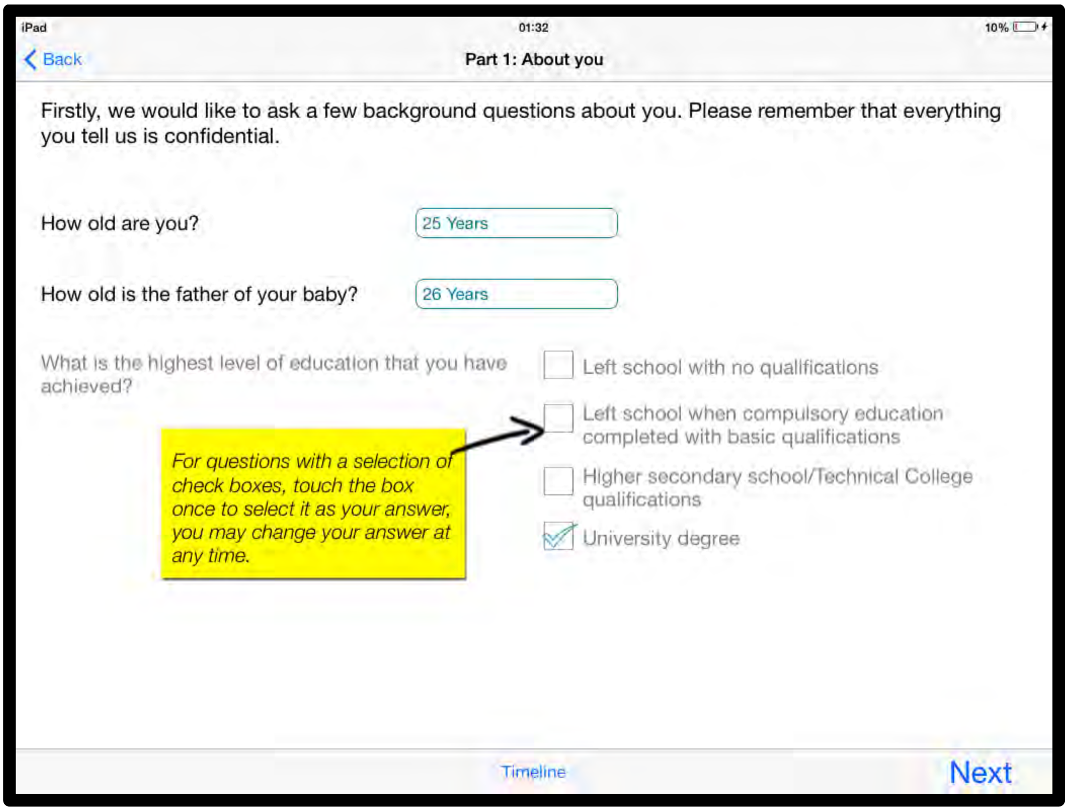
iPad
01:32
10%
Back
Part 1: About you
Firstly, we would like to ask a few background questions about you. Please remember that everything you tell us is confidential.
How old are you?
25 Years
How old is the father of your baby?
26 Years
What is the highest level of education that you have achieved?
Left school with no qualifications
Left school when compulsory education completed with basic qualifications
Higher secondary school/Technical College qualifications
University degree
For questions with a selection of check boxes, touch the box once to select it as your answer, you may change your answer at any time.
Timeline
Next

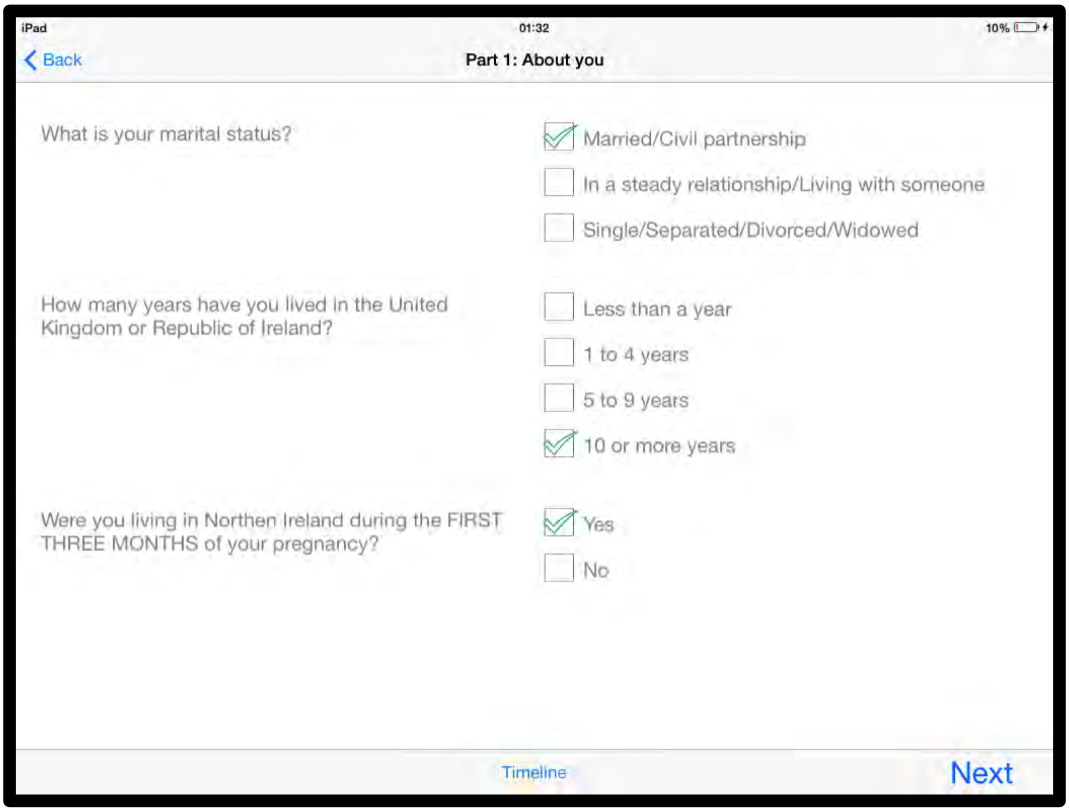
iPad
01:32
10%
Back
Part 1: About you
What is your marital status?
Married/Civil partnership
In a steady relationship/Living with someone
Single/Separated/Divorced/Widowed
How many years have you lived in the United Kingdom or Republic of Ireland?
Less than a year
1 to 4 years
5 to 9 years
10 or more years
Were you living in Northen Ireland during the FIRST THREE MONTHS of your pregnancy?
Yes
No
Timeline
Next

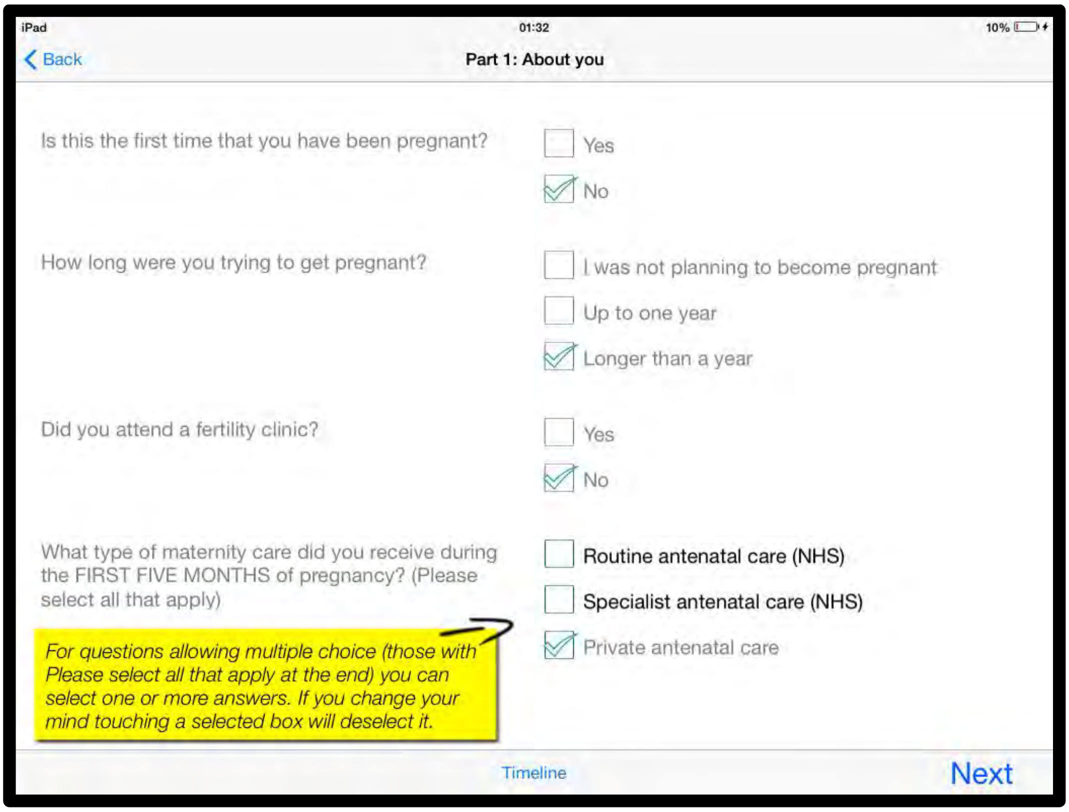
iPad
01:32
10%
Back
Part 1: About you
Is this the first time that you have been pregnant?
Yes
No
How long were you trying to get pregnant?
I was not planning to become pregnant
Up to one year
Longer than a year
Did you attend a fertility clinic?
Yes
No
What type of maternity care did you receive during the FIRST FIVE MONTHS of pregnancy? (Please select all that apply)
Routine antenatal care (NHS)
Specialist antenatal care (NHS)
Private antenatal care
For questions allowing multiple choice (those with Please select all that apply at the end) you can select one or more answers. If you change your mind touching a selected box will deselect it.
Timeline
Next

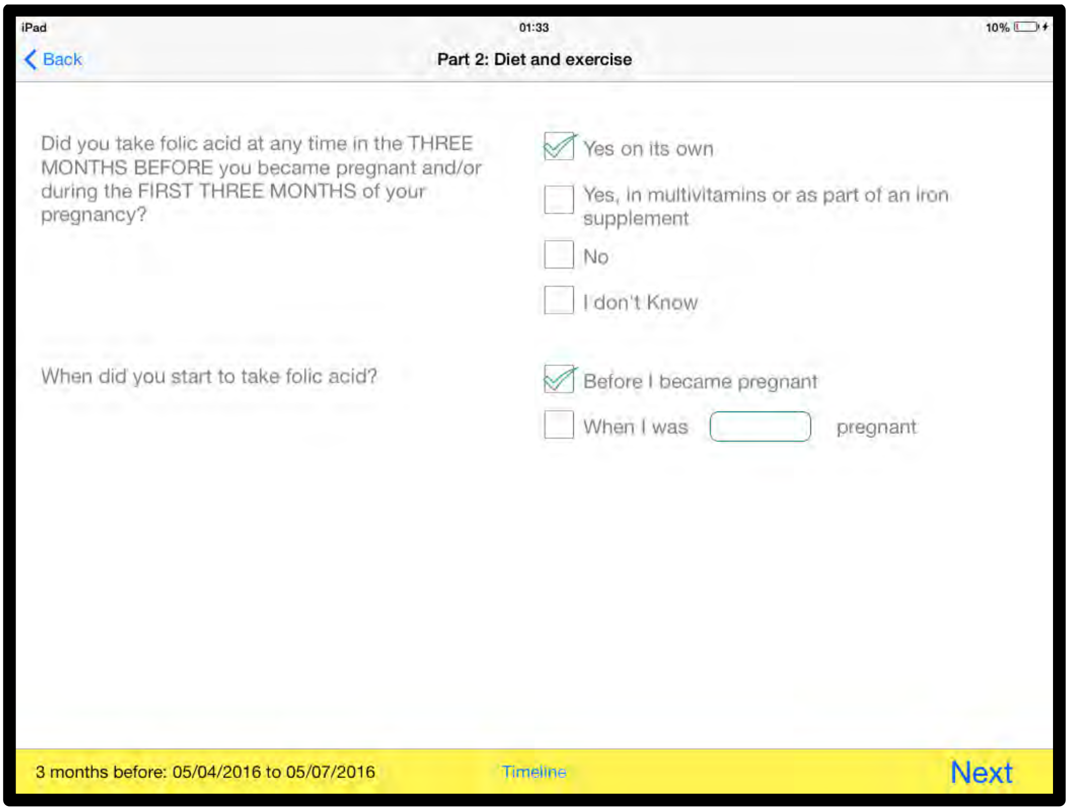

iPad 01:33 10%

Back

Part 2: Diet and exercise

Did you take folic acid at any time in the THREE MONTHS BEFORE you became pregnant and/or during the FIRST THREE MONTHS of your pregnancy?

- [x] Yes on its own
- [ ] Yes, in multivitamins or as part of an iron supplement
- [ ] No
- [ ] I don't Know

When did you start to take folic acid?

- [x] Before I became pregnant
- [ ] When I was ______ pregnant

3 months before: 05/04/2016 to 05/07/2016 Timeline Next

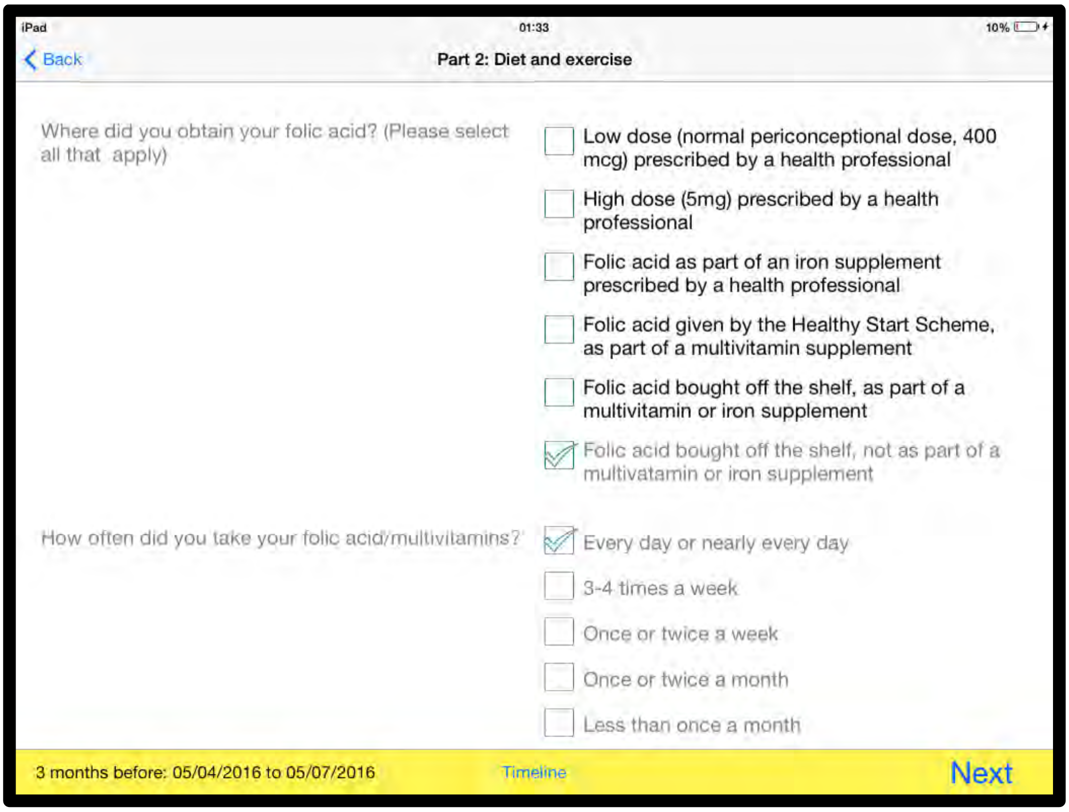

iPad 01:33 10%

Back

Part 2: Diet and exercise

Where did you obtain your folic acid? (Please select all that apply)

- [ ] Low dose (normal periconceptional dose, 400 mcg) prescribed by a health professional
- [ ] High dose (5mg) prescribed by a health professional
- [ ] Folic acid as part of an iron supplement prescribed by a health professional
- [ ] Folic acid given by the Healthy Start Scheme, as part of a multivitamin supplement
- [ ] Folic acid bought off the shelf, as part of a multivitamin or iron supplement
- [x] Folic acid bought off the shelf, not as part of a multivatamin or iron supplement

How often did you take your folic acid/multivitamins?

- [x] Every day or nearly every day
- [ ] 3-4 times a week
- [ ] Once or twice a week
- [ ] Once or twice a month
- [ ] Less than once a month

3 months before: 05/04/2016 to 05/07/2016 Timeline Next

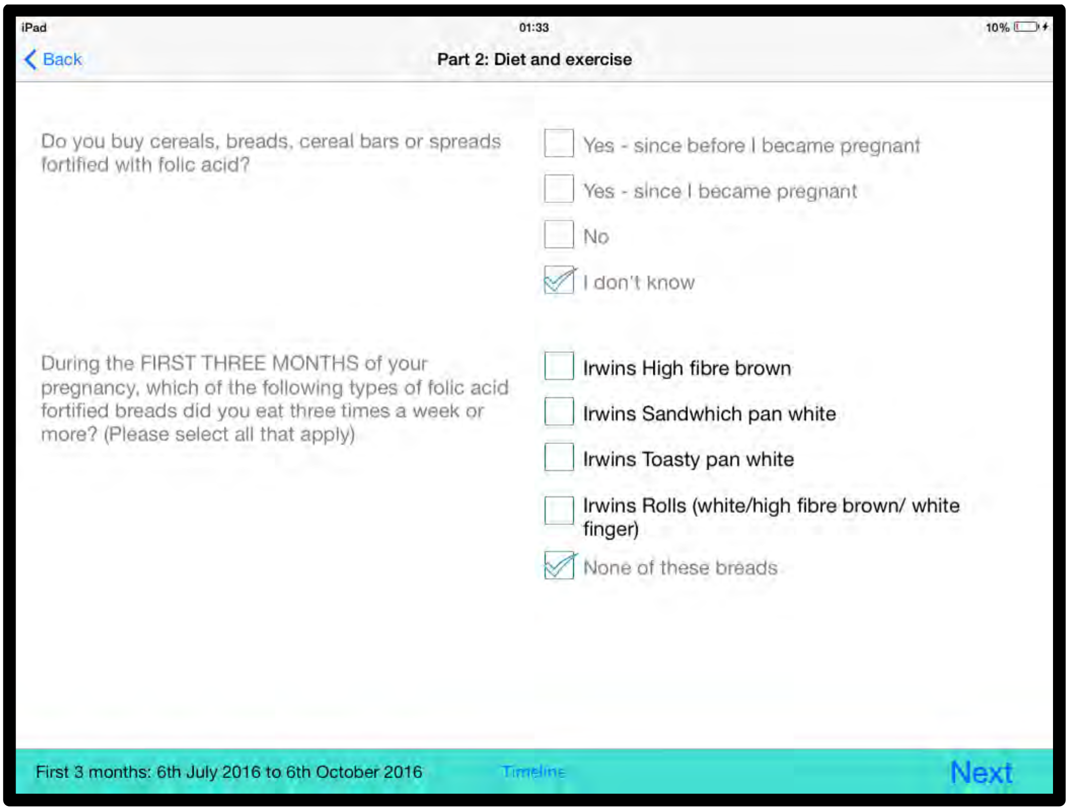

iPad 01:33 10%

Back

Part 2: Diet and exercise

Do you buy cereals, breads, cereal bars or spreads fortified with folic acid?

- [ ] Yes - since before I became pregnant
- [ ] Yes - since I became pregnant
- [ ] No
- [x] I don't know

During the FIRST THREE MONTHS of your pregnancy, which of the following types of folic acid fortified breads did you eat three times a week or more? (Please select all that apply)

- [ ] Irwins High fibre brown
- [ ] Irwins Sandwhich pan white
- [ ] Irwins Toasty pan white
- [ ] Irwins Rolls (white/high fibre brown/ white finger)
- [x] None of these breads

First 3 months: 6th July 2016 to 6th October 2016 Timeline Next

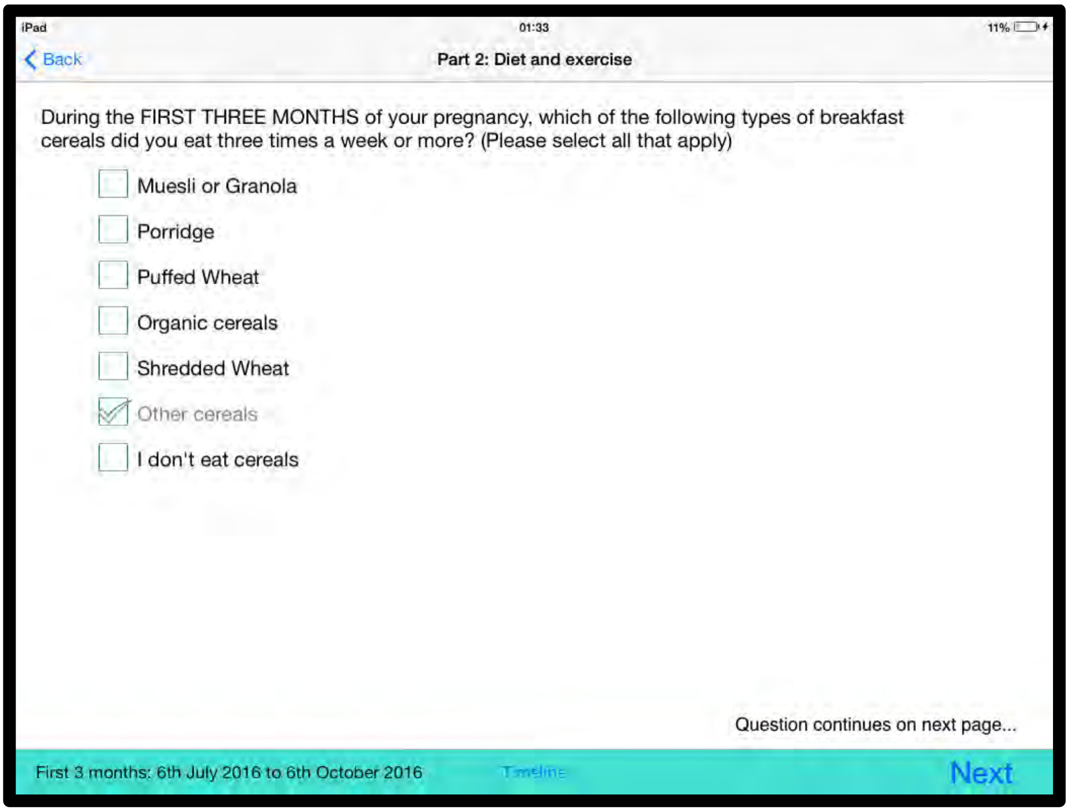

iPad 01:33 11%

Back

Part 2: Diet and exercise

During the FIRST THREE MONTHS of your pregnancy, which of the following types of breakfast cereals did you eat three times a week or more? (Please select all that apply)

- [ ] Muesli or Granola
- [ ] Porridge
- [ ] Puffed Wheat
- [ ] Organic cereals
- [ ] Shredded Wheat
- [x] Other cereals
- [ ] I don't eat cereals

Question continues on next page...

First 3 months: 6th July 2016 to 6th October 2016 Timeline Next

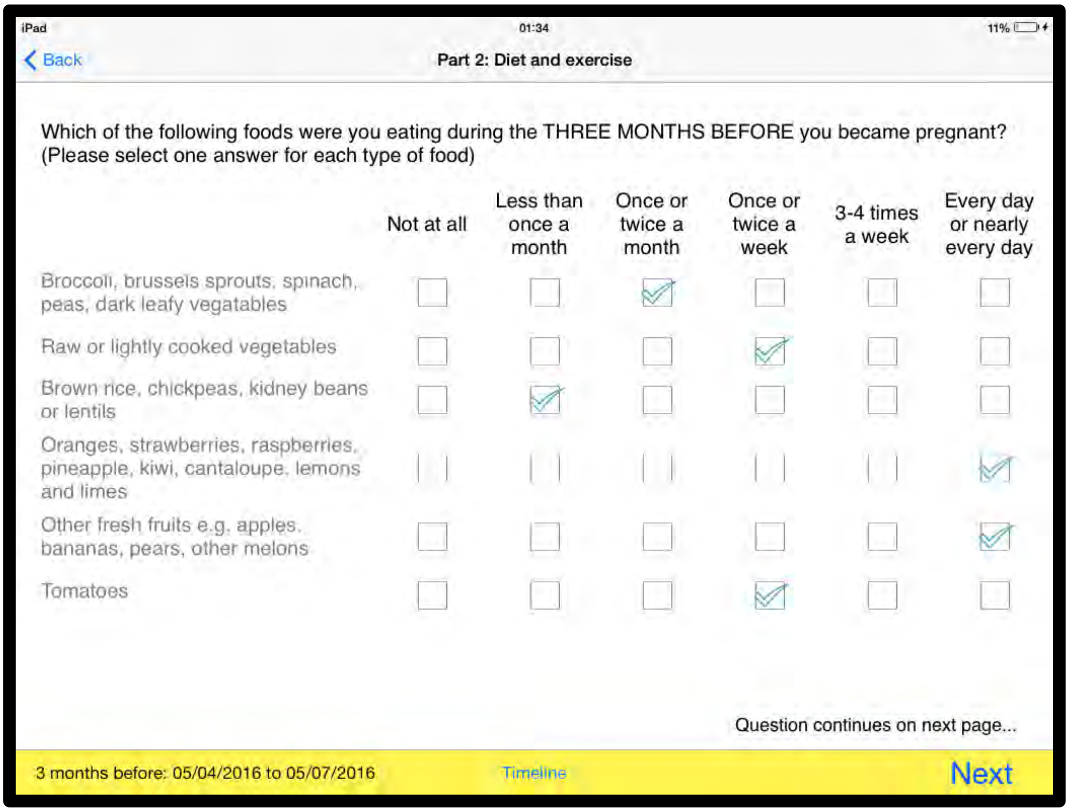

iPad 01:34 11%

Back

Part 2: Diet and exercise

Which of the following foods were you eating during the THREE MONTHS BEFORE you became pregnant? (Please select one answer for each type of food)

	Not at all	Less than once a month	Once or twice a month	Once or twice a week	3-4 times a week	Every day or nearly every day
Broccoli, brussels sprouts, spinach, peas, dark leafy vegatables			✓			
Raw or lightly cooked vegetables				✓		
Brown rice, chickpeas, kidney beans or lentils		✓				
Oranges, strawberries, raspberries, pineapple, kiwi, cantaloupe, lemons and limes						✓
Other fresh fruits e.g. apples, bananas, pears, other melons						✓
Tomatoes				✓		

Question continues on next page...

3 months before: 05/04/2016 to 05/07/2016 Timeline Next

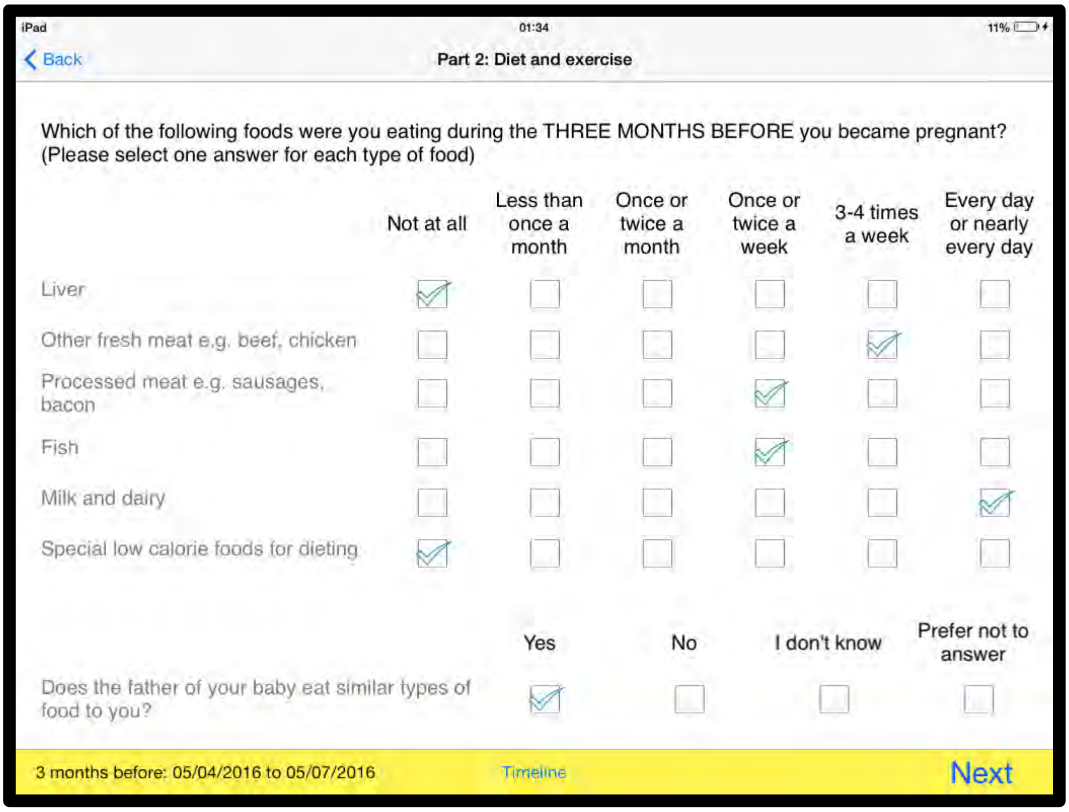

iPad 01:34 11%

Back

Part 2: Diet and exercise

Which of the following foods were you eating during the THREE MONTHS BEFORE you became pregnant? (Please select one answer for each type of food)

	Not at all	Less than once a month	Once or twice a month	Once or twice a week	3-4 times a week	Every day or nearly every day
Liver	✓					
Other fresh meat e.g. beef, chicken					✓	
Processed meat e.g. sausages, bacon				✓		
Fish				✓		
Milk and dairy						✓
Special low calorie foods for dieting	✓					

	Yes	No	I don't know	Prefer not to answer
Does the father of your baby eat similar types of food to you?	✓			

3 months before: 05/04/2016 to 05/07/2016 Timeline Next

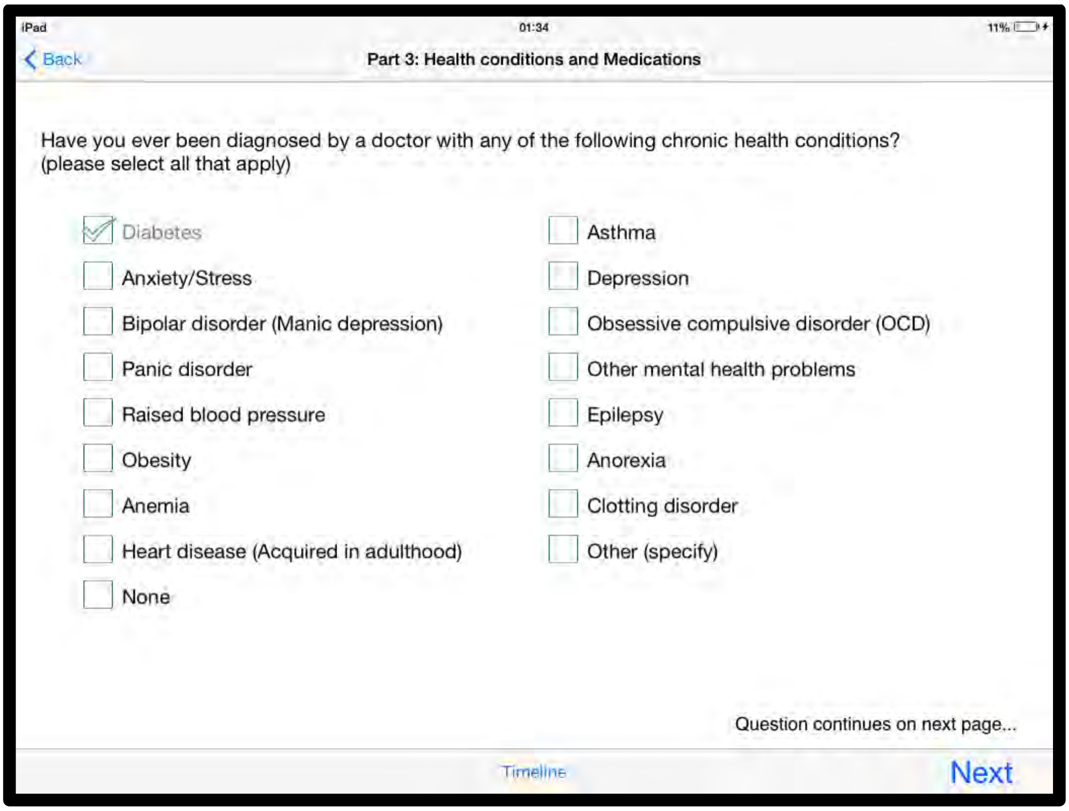
iPad
01:34
11%
Back
Part 3: Health conditions and Medications
Have you ever been diagnosed by a doctor with any of the following chronic health conditions? (please select all that apply)
Diabetes
Asthma
Anxiety/Stress
Depression
Bipolar disorder (Manic depression)
Obsessive compulsive disorder (OCD)
Panic disorder
Other mental health problems
Raised blood pressure
Epilepsy
Obesity
Anorexia
Anemia
Clotting disorder
Heart disease (Acquired in adulthood)
Other (specify)
None
Question continues on next page...
Timeline
Next

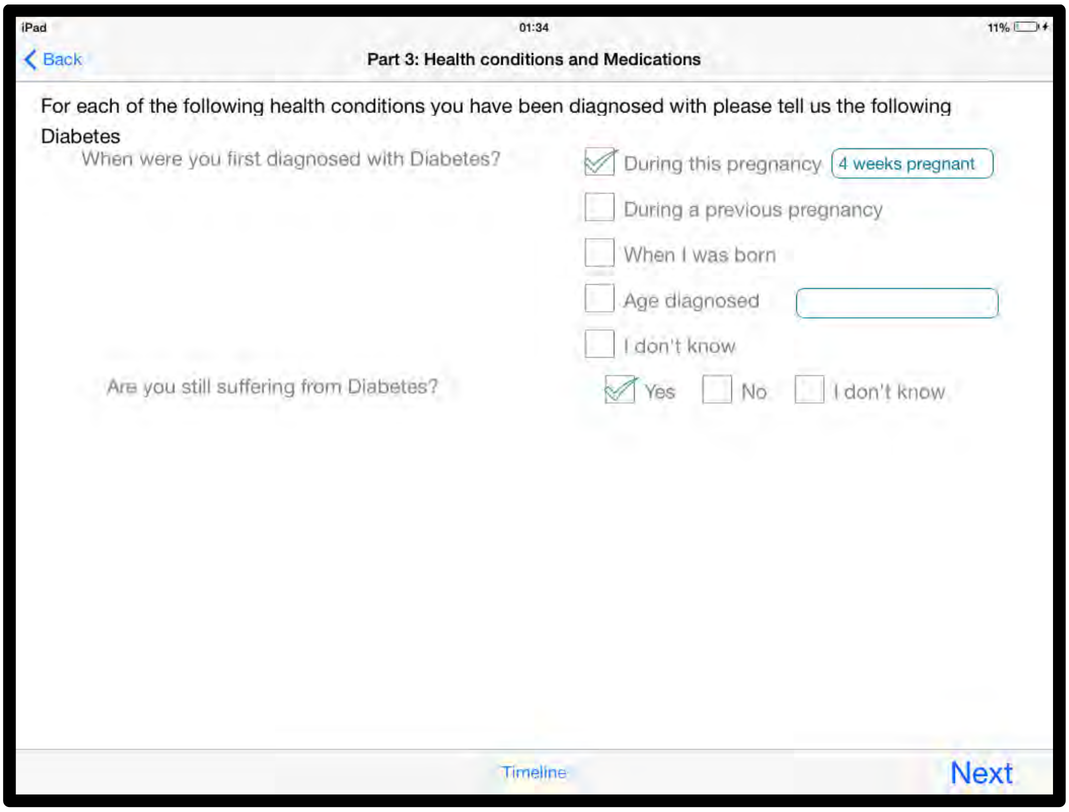
iPad
01:34
11%
Back
Part 3: Health conditions and Medications
For each of the following health conditions you have been diagnosed with please tell us the following
Diabetes
When were you first diagnosed with Diabetes?
During this pregnancy
4 weeks pregnant
During a previous pregnancy
When I was born
Age diagnosed
I don't know
Are you still suffering from Diabetes?
Yes
No
I don't know
Timeline
Next

iPad 01:34 11%

Back **Part 3: Health conditions and Medications**

Has the father of your baby ever been diagnosed by a doctor with any of the following chronic health conditions? (please select all that apply)

- [ ] Diabetes
- [ ] Asthma
- [ ] Anxiety/Stress
- [ ] Depression
- [ ] Bipolar disorder (Manic depression)
- [ ] Obsessive compulsive disorder (OCD)
- [ ] Panic disorder
- [ ] Other mental health problems
- [ ] Raised blood pressure
- [ ] Epilepsy
- [ ] Obesity
- [ ] Anorexia
- [ ] Anemia
- [ ] Clotting disorder
- [ ] Heart disease (Aquired in adulthood)
- [ ] Other (specify)
- [ ] I don't know
- [x] None
- [ ] Prefer not to answer

Timeline Next

iPad 01:34 11%

Back **Part 3: Health conditions and Medications**

During the FIRST THREE MONTHS of your pregnancy were you diagnosed with any of the following infections? (please select all that apply)

- [ ] Rubella (German measles)
- [ ] Influenza
- [ ] Varicella (Chicken pox)
- [ ] Parvovirus (Slapped cheek)
- [ ] Toxoplasmosis
- [ ] Cytomegalovirus
- [x] Kidney/Urinary infection
- [ ] Vaginal infection/Thrush
- [ ] Other (specify)
- [ ] None

First 3 months: 6th July 2016 to 6th October 2016 Timeline Next

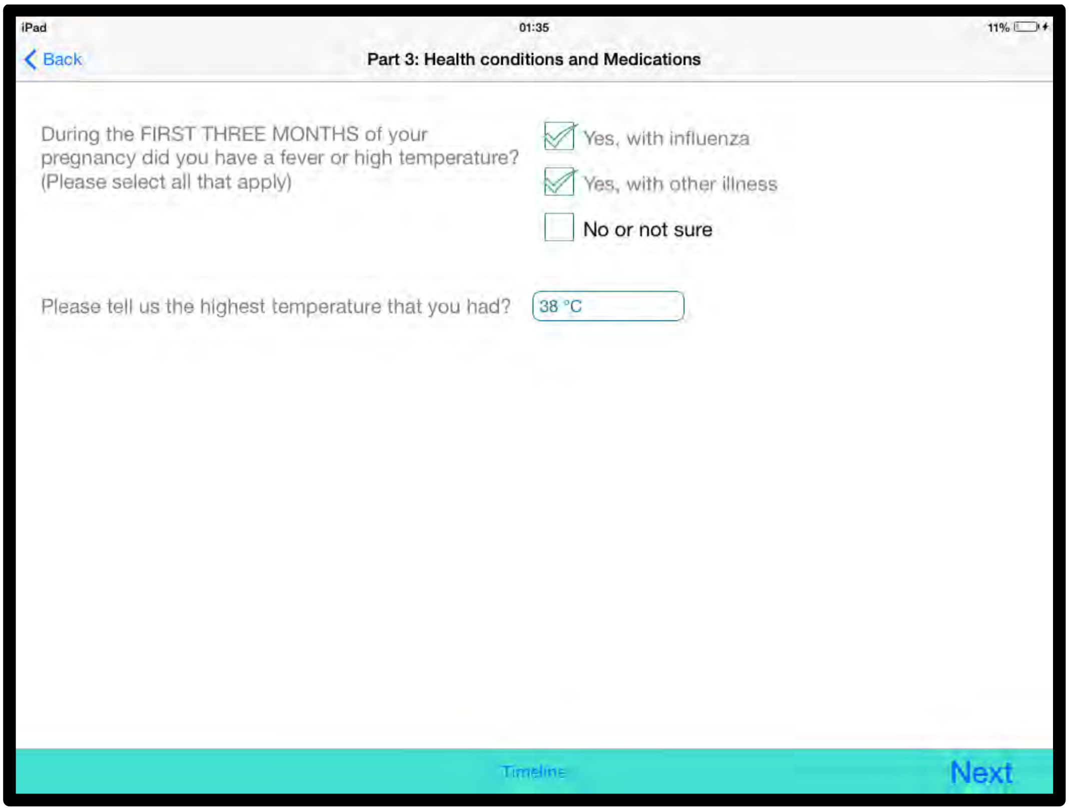

iPad 01:35 11%

Back

Part 3: Health conditions and Medications

During the FIRST THREE MONTHS of your pregnancy did you have a fever or high temperature? (Please select all that apply)

- [x] Yes, with influenza
- [x] Yes, with other illness
- [ ] No or not sure

Please tell us the highest temperature that you had? 38 °C

Timeline Next

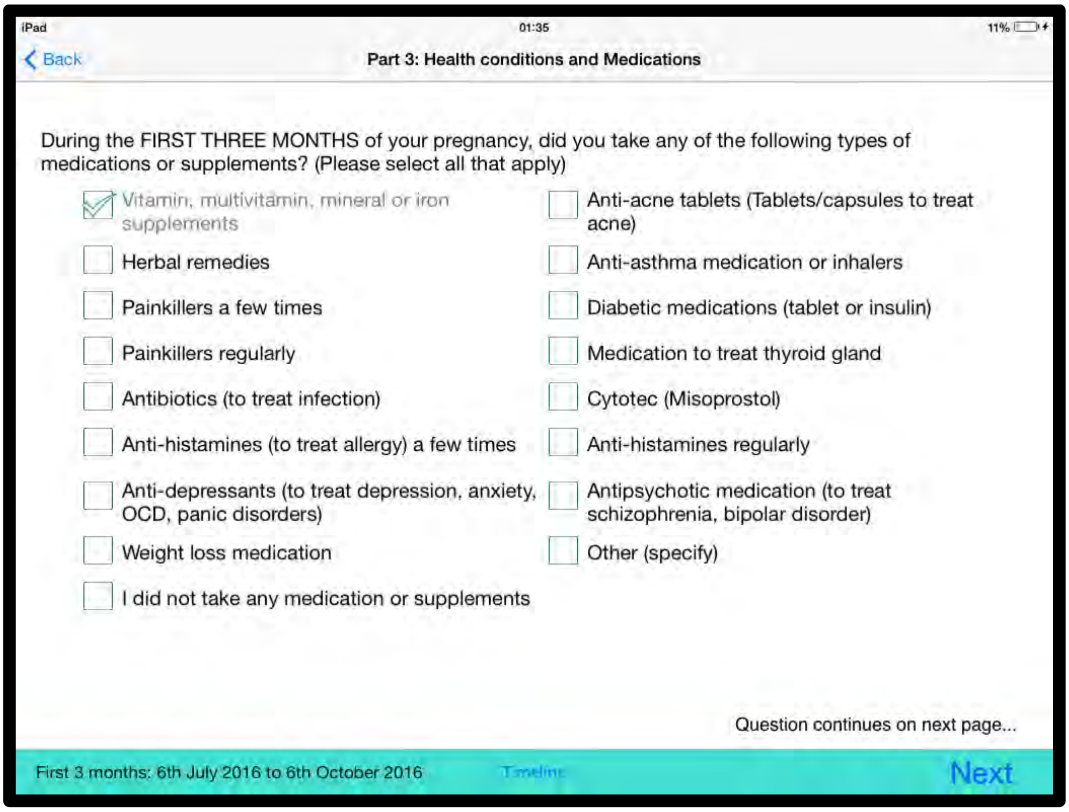

iPad 01:35 11%

Back

Part 3: Health conditions and Medications

During the FIRST THREE MONTHS of your pregnancy, did you take any of the following types of medications or supplements? (Please select all that apply)

- [x] Vitamin, multivitamin, mineral or iron supplements
- [ ] Anti-acne tablets (Tablets/capsules to treat acne)
- [ ] Herbal remedies
- [ ] Anti-asthma medication or inhalers
- [ ] Painkillers a few times
- [ ] Diabetic medications (tablet or insulin)
- [ ] Painkillers regularly
- [ ] Medication to treat thyroid gland
- [ ] Antibiotics (to treat infection)
- [ ] Cytotec (Misoprostol)
- [ ] Anti-histamines (to treat allergy) a few times
- [ ] Anti-histamines regularly
- [ ] Anti-depressants (to treat depression, anxiety, OCD, panic disorders)
- [ ] Antipsychotic medication (to treat schizophrenia, bipolar disorder)
- [ ] Weight loss medication
- [ ] Other (specify)
- [ ] I did not take any medication or supplements

Question continues on next page...

First 3 months: 6th July 2016 to 6th October 2016 Timeline Next

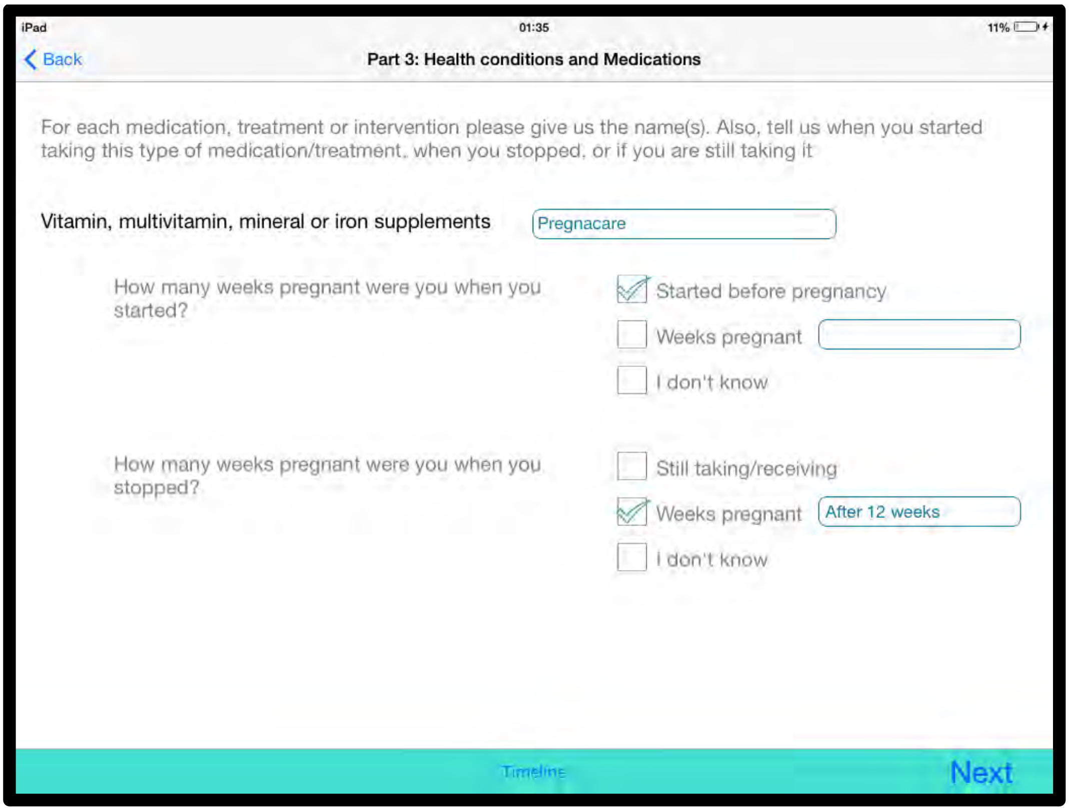

iPad 01:35 11%

Back **Part 3: Health conditions and Medications**

For each medication, treatment or intervention please give us the name(s). Also, tell us when you started taking this type of medication/treatment, when you stopped, or if you are still taking it

Vitamin, multivitamin, mineral or iron supplements: Pregnacare

How many weeks pregnant were you when you started?
- [x] Started before pregnancy
- [ ] Weeks pregnant
- [ ] I don't know

How many weeks pregnant were you when you stopped?
- [ ] Still taking/receiving
- [x] Weeks pregnant: After 12 weeks
- [ ] I don't know

Timeline Next

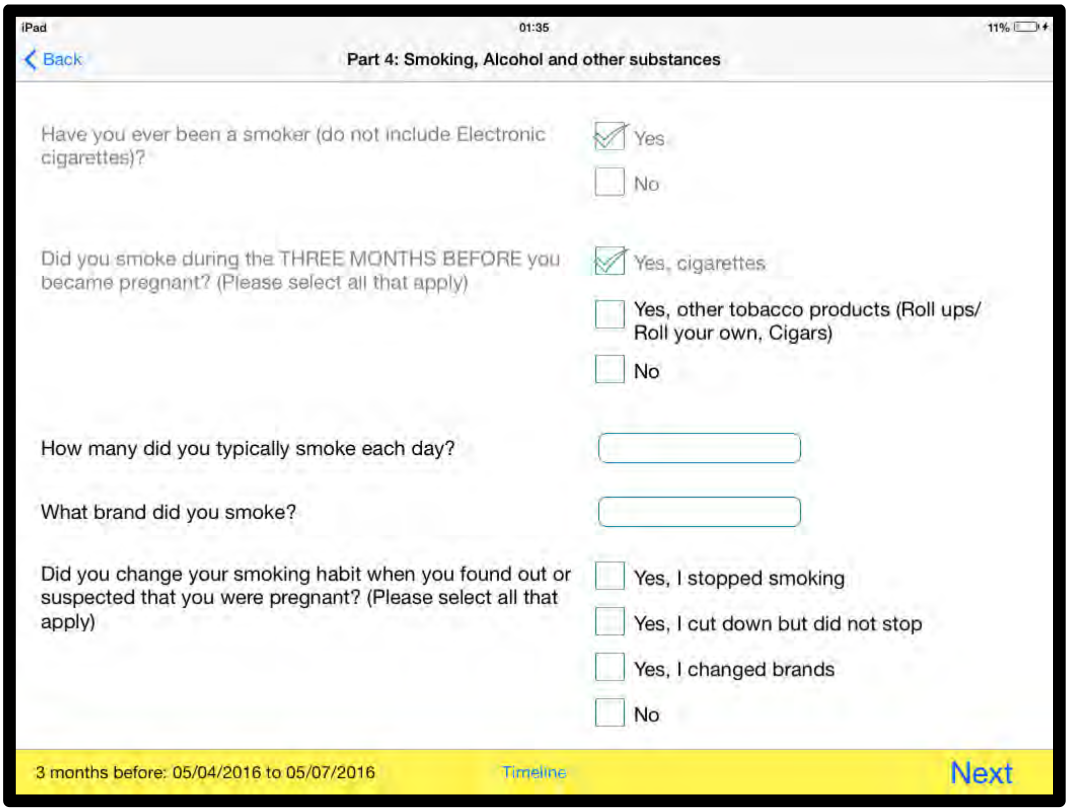

iPad 01:35 11%

Back **Part 4: Smoking, Alcohol and other substances**

Have you ever been a smoker (do not include Electronic cigarettes)?
- [x] Yes
- [ ] No

Did you smoke during the THREE MONTHS BEFORE you became pregnant? (Please select all that apply)
- [x] Yes, cigarettes
- [ ] Yes, other tobacco products (Roll ups/ Roll your own, Cigars)
- [ ] No

How many did you typically smoke each day?

What brand did you smoke?

Did you change your smoking habit when you found out or suspected that you were pregnant? (Please select all that apply)
- [ ] Yes, I stopped smoking
- [ ] Yes, I cut down but did not stop
- [ ] Yes, I changed brands
- [ ] No

3 months before: 05/04/2016 to 05/07/2016 Timeline Next

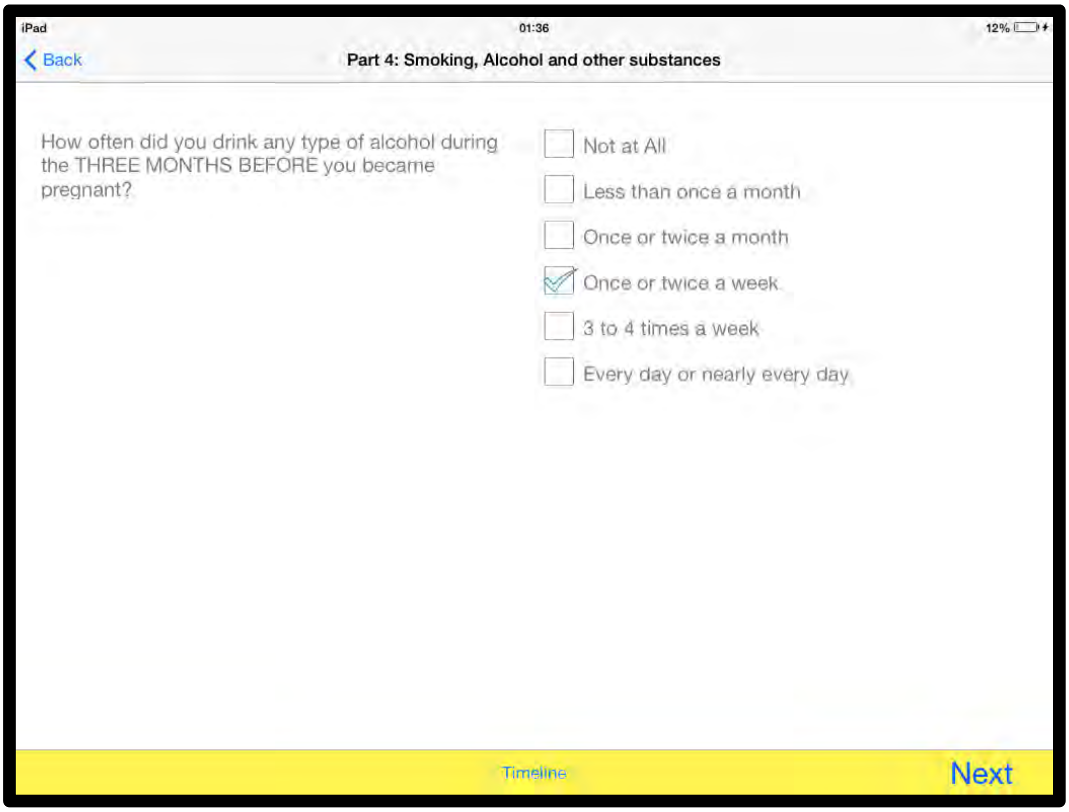
iPad 01:36 12%

Back

Part 4: Smoking, Alcohol and other substances

How often did you drink any type of alcohol during the THREE MONTHS BEFORE you became pregnant?

- [ ] Not at All
- [ ] Less than once a month
- [ ] Once or twice a month
- [x] Once or twice a week
- [ ] 3 to 4 times a week
- [ ] Every day or nearly every day

Timeline Next

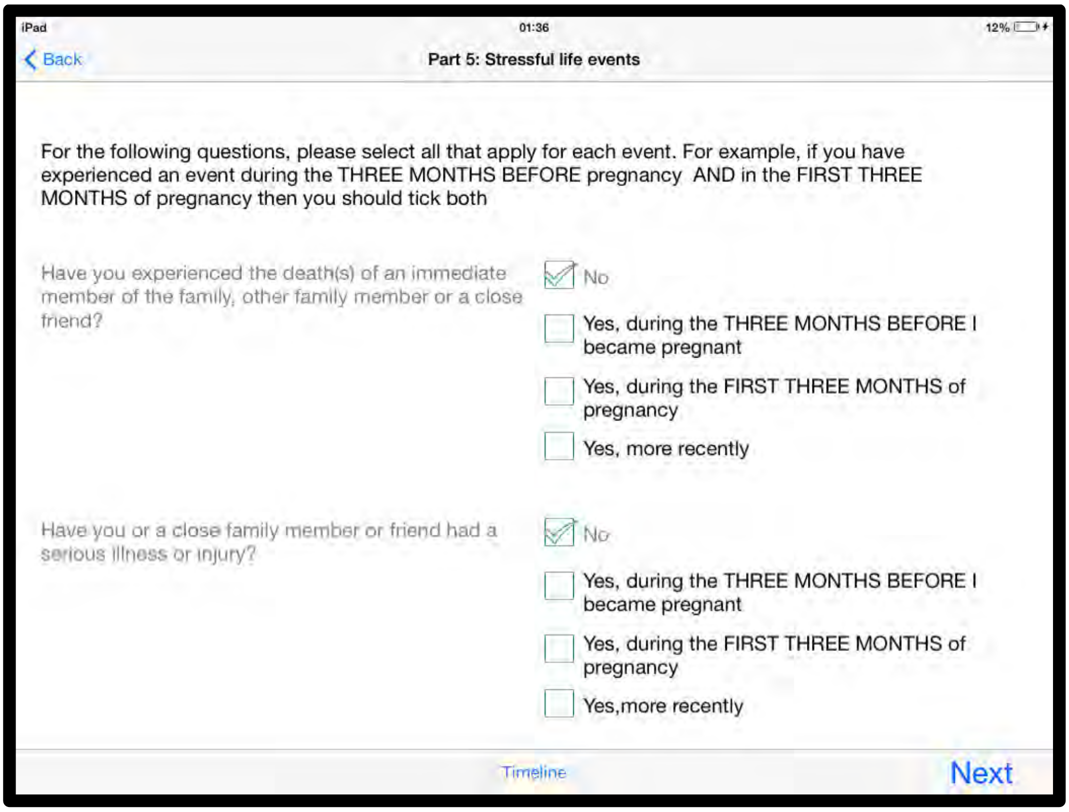
iPad 01:36 12%

Back

Part 5: Stressful life events

For the following questions, please select all that apply for each event. For example, if you have experienced an event during the THREE MONTHS BEFORE pregnancy AND in the FIRST THREE MONTHS of pregnancy then you should tick both

Have you experienced the death(s) of an immediate member of the family, other family member or a close friend?

- [x] No
- [ ] Yes, during the THREE MONTHS BEFORE I became pregnant
- [ ] Yes, during the FIRST THREE MONTHS of pregnancy
- [ ] Yes, more recently

Have you or a close family member or friend had a serious illness or injury?

- [x] No
- [ ] Yes, during the THREE MONTHS BEFORE I became pregnant
- [ ] Yes, during the FIRST THREE MONTHS of pregnancy
- [ ] Yes,more recently

Timeline Next

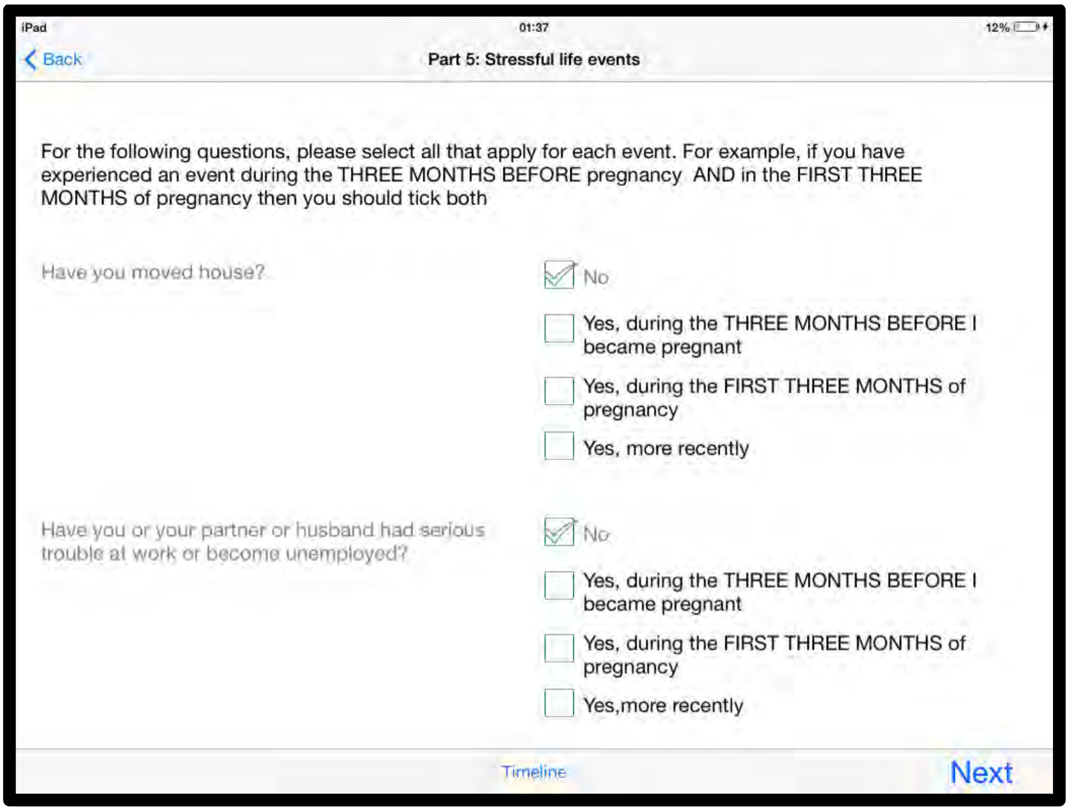

iPad 01:37 12%

Back

Part 5: Stressful life events

For the following questions, please select all that apply for each event. For example, if you have experienced an event during the THREE MONTHS BEFORE pregnancy AND in the FIRST THREE MONTHS of pregnancy then you should tick both

Have you moved house?

- [x] No
- [ ] Yes, during the THREE MONTHS BEFORE I became pregnant
- [ ] Yes, during the FIRST THREE MONTHS of pregnancy
- [ ] Yes, more recently

Have you or your partner or husband had serious trouble at work or become unemployed?

- [x] No
- [ ] Yes, during the THREE MONTHS BEFORE I became pregnant
- [ ] Yes, during the FIRST THREE MONTHS of pregnancy
- [ ] Yes,more recently

Timeline Next

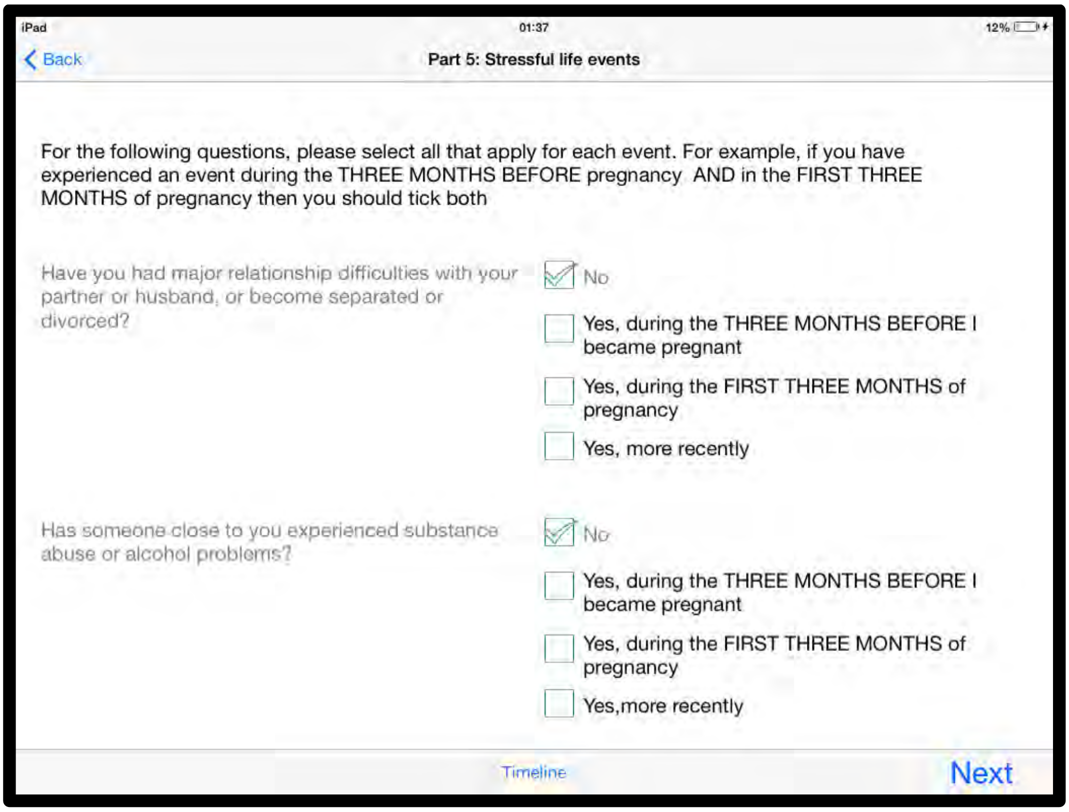

iPad 01:37 12%

Back

Part 5: Stressful life events

For the following questions, please select all that apply for each event. For example, if you have experienced an event during the THREE MONTHS BEFORE pregnancy AND in the FIRST THREE MONTHS of pregnancy then you should tick both

Have you had major relationship difficulties with your partner or husband, or become separated or divorced?

- [x] No
- [ ] Yes, during the THREE MONTHS BEFORE I became pregnant
- [ ] Yes, during the FIRST THREE MONTHS of pregnancy
- [ ] Yes, more recently

Has someone close to you experienced substance abuse or alcohol problems?

- [x] No
- [ ] Yes, during the THREE MONTHS BEFORE I became pregnant
- [ ] Yes, during the FIRST THREE MONTHS of pregnancy
- [ ] Yes,more recently

Timeline Next

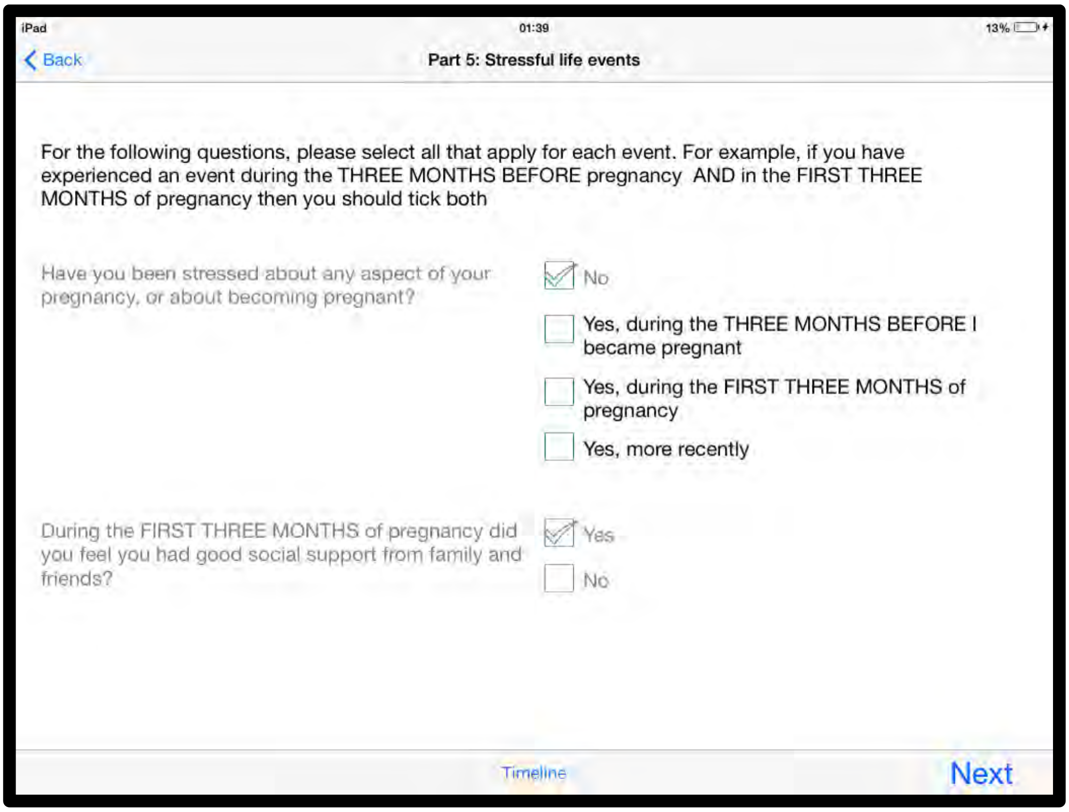
iPad
01:39
13%
Back
Part 5: Stressful life events
For the following questions, please select all that apply for each event. For example, if you have experienced an event during the THREE MONTHS BEFORE pregnancy AND in the FIRST THREE MONTHS of pregnancy then you should tick both
Have you been stressed about any aspect of your pregnancy, or about becoming pregnant?
No
Yes, during the THREE MONTHS BEFORE I became pregnant
Yes, during the FIRST THREE MONTHS of pregnancy
Yes, more recently
During the FIRST THREE MONTHS of pregnancy did you feel you had good social support from family and friends?
Yes
No
Timeline
Next

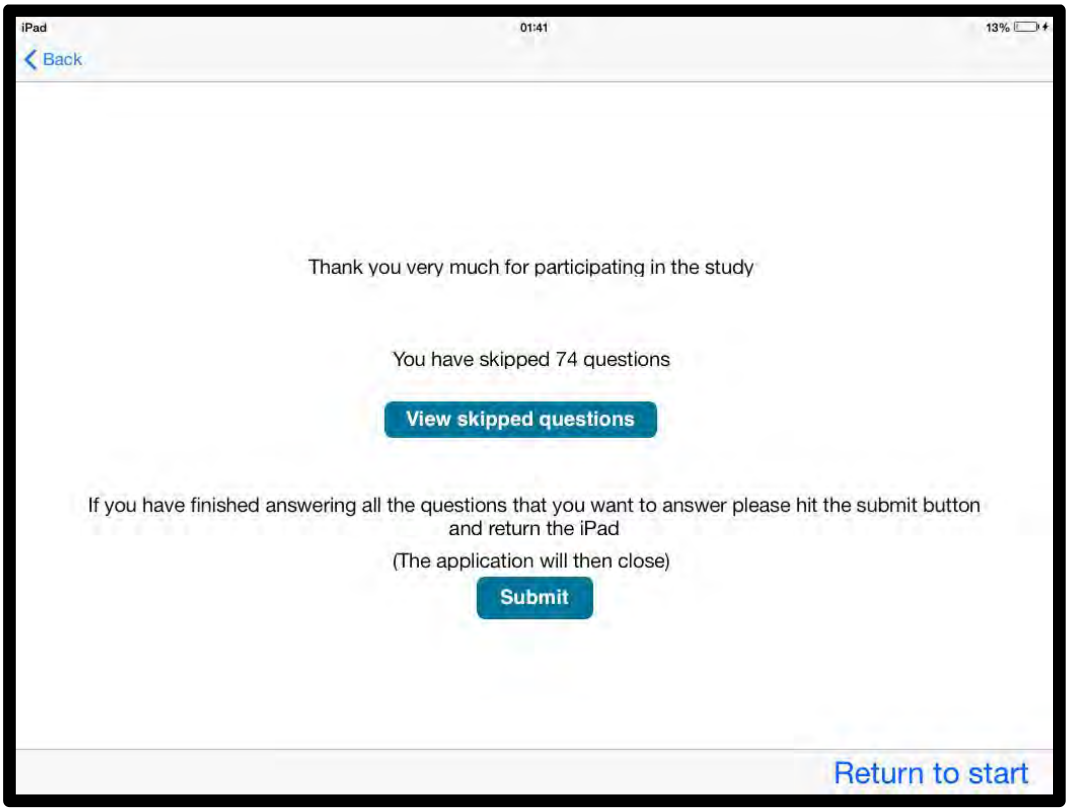
iPad
01:41
13%
Back
Thank you very much for participating in the study
You have skipped 74 questions
View skipped questions
If you have finished answering all the questions that you want to answer please hit the submit button and return the iPad
(The application will then close)
Submit
Return to start
